# S‐ketamine Alleviates Neuroinflammation and Attenuates Lipopolysaccharide‐Induced Depression Via Targeting SIRT2

**DOI:** 10.1002/advs.202416481

**Published:** 2025-04-02

**Authors:** Cong Lin, Xiaoxuan Zhou, Mingqi Li, Cong Zhang, Haojiang Zhai, Haohong Li, Hongshuang Wang, Xiaohui Wang

**Affiliations:** ^1^ Laboratory of Chemical Biology Changchun Institute of Applied Chemistry Chinese Academy of Sciences Changchun Jilin 130022 China; ^2^ School of Applied Chemistry and Engineering University of Science and Technology of China Hefei Anhui 230026 China; ^3^ The MOE Frontier Research Center of Brain and Brain‐Machine Integration Zhejiang University School of Brain Science and Brain Medicine Hangzhou Zhejiang 310058 China; ^4^ State Key Lab of Brain‐Machine Intelligence Zhejiang University Hangzhou 311121 China

**Keywords:** Depression, Neuroinflammation, R‐ketamine, SIRT2, S‐ketamine

## Abstract

Depression, a pervasive mental health condition, has increasingly been linked to neuroinflammation, as evidenced by elevated levels of pro‐inflammatory markers such as TNF‐α and IL‐1β observed in patients, which underscores the role of inflammation in its pathophysiology. This study investigates the differential effects of S‐ketamine (S‐KET) and R‐ketamine (R‐KET) on inflammation‐induced depression using a lipopolysaccharide (LPS)‐induced mouse model. Results showed that S‐KET, but not R‐KET, significantly alleviated depressive‐like behaviors and reduced levels of pro‐inflammatory factors in the medial prefrontal cortex (mPFC). Activity‐based protein profiling identified SIRT2 as a key intracellular target of S‐KET, with direct binding observed at the Q167 residue, whereas R‐KET showed no such binding. S‐KET enhanced SIRT2 interaction with NF‐κB subunit p65, reducing its acetylation and suppressing pro‐inflammatory gene expression, effects not seen with R‐KET. In vitro studies with RNA interference and the SIRT2 inhibitor AK‐7, along with in vivo pharmacological blockade, confirmed that SIRT2 is crucial for the anti‐inflammatory and antidepressant actions of S‐KET. These findings suggest that SIRT2 mediates the therapeutic effects of S‐KET, highlighting its potential as a target for treating inflammation‐associated depression. This study provides novel insights into the stereospecific actions of ketamine enantiomers and the promise of targeting SIRT2 for neuroinflammatory depression.

## Graphical abstract

S‐ketamine binds directly to SIRT2, facilitating its interaction with NF‐κB p65. This interaction reduces the acetylation of NF‐κB p65 and inhibits its activation, thereby exerting anti‐inflammatory and antidepressant effects. Created by BioRender.

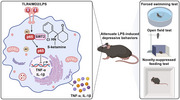

## 1 Introduction

Depression is a prevalent mental health condition that affects ≈280 million people worldwide, accounting for ≈ 4% of the global population. Characterized by persistent low mood, loss of interest or pleasure in daily activities, and cognitive impairments, depression significantly reduces quality of life.^[^ ^1^, ^2^, ^3^ ^]^ Furthermore, depression contributes to ≈ 700000 deaths annually, as reported by the World Health Organization (WHO).^[^ ^4^ ^]^ Recent meta‐analyses and large cohort studies have increasingly highlighted the role of neuroinflammation in the pathophysiology of depression, with chronic low‐grade inflammation commonly observed in patients.^[^ ^5^, ^6^ ^]^ Elevated levels of pro‐inflammatory cytokines, such as interleukin‐1β (IL‐1β) and tumor necrosis factor‐alpha (TNF‐α), in the blood of patients with depression, which points to the involvement of neuroinflammatory processes in the pathophysiology of depressive symptoms.^[^ ^7^, ^8^, ^9^, ^10^ ^]^ Microglial activation and neuroinflammation are crucial for synaptic pruning and the modulation of neuroplasticity, which are essential processes for maintaining healthy brain function. In depression, dysregulated microglial activation and neuroinflammation can result in excessive synaptic pruning and impaired neuroplasticity, ultimately leading to dysfunction in the neural circuits involved in mood regulation.^[^ ^11^, ^12^ ^]^ Moreover, neuroinflammation could lead to disruptions in serotonin and dopamine metabolism, both of which are critical to mood regulation.^[^ ^13^, ^14^, ^15^ ^]^

S‐ketamine (S‐KET), an N‐methyl‐D‐aspartate (NMDA) receptor antagonist, was approved by the Food and Drug Administration (FDA) in 2019 for the treatment of major depressive disorder (MDD) and treatment‐resistant depression (TRD).^[^ ^16^, ^17^, ^18^ ^]^ Long before its use in MDD and TRD, ketamine was primarily used as a surgical anesthetic. Notably, ketamine also exhibits significant anti‐inflammatory activity beyond its traditional anesthetic and analgesic roles, as demonstrated in various animal models and clinical settings for both acute and chronic inflammatory conditions, including depression.^[^ ^19^ ^]^ Despite these promising effects, the molecular target of ketamine for inhibiting neuroinflammation remains underexplored. Given that the enantiomers of ketamine, S‐KET and R‐KET, exhibit distinct pharmacological activities,^[^ ^20^ ^]^ it is important to systematically explore their differential effects on neuroinflammation. In this study, SIRT2 was identified as the direct target of S‐KET. S‐KET enhanced the interaction between SIRT2 and NF‐κB subunit p65, reducing its acetylation, inhibiting NF‐κB activation, and thereby suppressing lipopolysaccharide (LPS)‐induced pro‐inflammatory factors. In contrast, R‐KET neither bound to SIRT2 nor influenced p65 acetylation or pro‐inflammatory factor production. RNA interference and pharmacological approaches confirmed SIRT2 as the mediator of S‐KET's anti‐inflammatory effects at the cellular level, with further in vivo validation identifying SIRT2 as key to S‐KET's antidepressant effect. These findings suggest that SIRT2 is a promising intracellular target for neuroinflammation and inflammation‐induced depression.

## 2 Results

### 2.1 The Effects of S‐KET and R‐KET on LPS‐Induced Depression and Neuroinflammation

**Figure 1:**
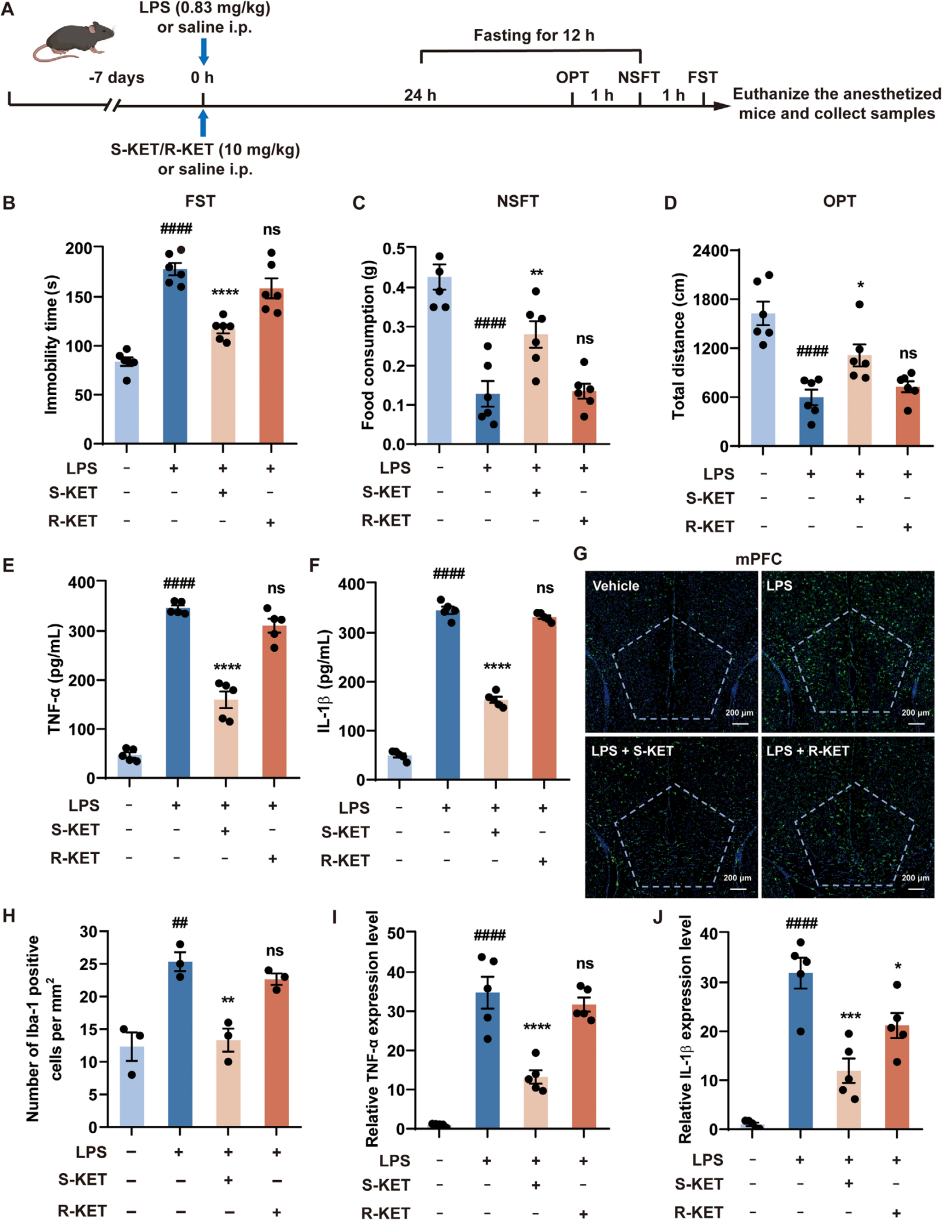
S‐KET alleviates LPS‐induced depressive‐like behaviors and neuroinflammation. A) Schematic illustration of the experiment and timeline. B) Immobility time of FST (n = 6 per group). C) Food consumption of NSFT (n = 6 per group). D) Total distance of OPT (n = 6 per group). Serum samples and mPFC brain regions were collected following the behavioral tests. E,F) The pro‐inflammatory factors TNF‐α (E) and IL‐1β (F) in the serum were measured by ELISA (n = 5). G) Representative immunofluorescent images of the microglia activation marker Iba‐1 in the mPFC (n = 3). Scale bar: 200 µm. H) Quantification of Iba‐1^+^ microglial numbers shown in panel (G). I,J) TNF‐α (I) and IL‐1β (J) mRNA expression in the mPFC were quantified by qPCR (n = 5). Data were presented as mean ± SEM and analyzed by one‐way ANOVA. # was used for comparisons with the vehicle group, * was used for comparisons with the LPS group. *p < 0.05, ##/**p < 0.01, ***p < 0.001, ####/****p < 0.0001. ns denoted *p* ≥ 0.05.

To evaluate the effects of S‐KET and R‐KET on inflammation‐induced depressive‐like behavior, we established a mouse model of depression using LPS‐induced inflammation. Mice were administered LPS (0.83 mg kg^−1^) intraperitoneally^[^ ^21^, ^22^, ^23^ ^]^ and subsequently subjected to a series of behavioral tests, including the forced swimming test (FST), novelty‐suppressed feeding test (NSFT), and open field test (OFT) (**Figure** **1**A). LPS‐treated mice exhibited increased immobility in the FST (Figure 1B), reduced food intake in the NSFT (Figure 1C), and decreased total distance traveled in the OFT (Figure 1D), indicating clear depressive‐like behavior. Treatment with S‐KET (10 mg kg^−1^) significantly improved these depressive‐like behaviors compared to the LPS group, while the same dose of R‐KET showed no effect (Figure 1B–D), suggesting that S‐KET is more effective than R‐KET in alleviating LPS‐induced depressive‐like behavior in mice.

Following the behavioral tests, serum levels of pro‐inflammatory factors were measured using enzyme‐linked immunosorbent assay (ELISA). LPS treatment significantly elevated TNF‐α (Figure 1E) and IL‐1β (Figure 1F), and S‐KET administration markedly reduced these levels, while R‐KET had no inhibitory effect (Figure 1E,F), indicating that S‐KET possesses stronger anti‐inflammatory properties. To further explore the effects in the brain, we isolated the medial prefrontal cortex (mPFC) and performed immunofluorescence staining for microglial activation marker Iba‐1. LPS treatment induced significant microglial activation in the mPFC, which was significantly reduced by S‐KET; R‐KET, however, showed no such effect (Figure 1G,H). Additionally, qPCR analysis revealed that LPS significantly increased the mRNA expression of TNF‐α (Figure 1I) and IL‐1β (Figure 1J) in the mPFC, which was effectively suppressed by S‐KET, whereas R‐KET had no impact. Taken together, these findings indicate that S‐KET has more potent anti‐inflammatory and antidepressant effects compared to R‐KET in inflammation‐induced depression.

### 2.2 Target Fishing and Validation of SIRT2 as a Direct Target of S‐KET

**Figure 2:**
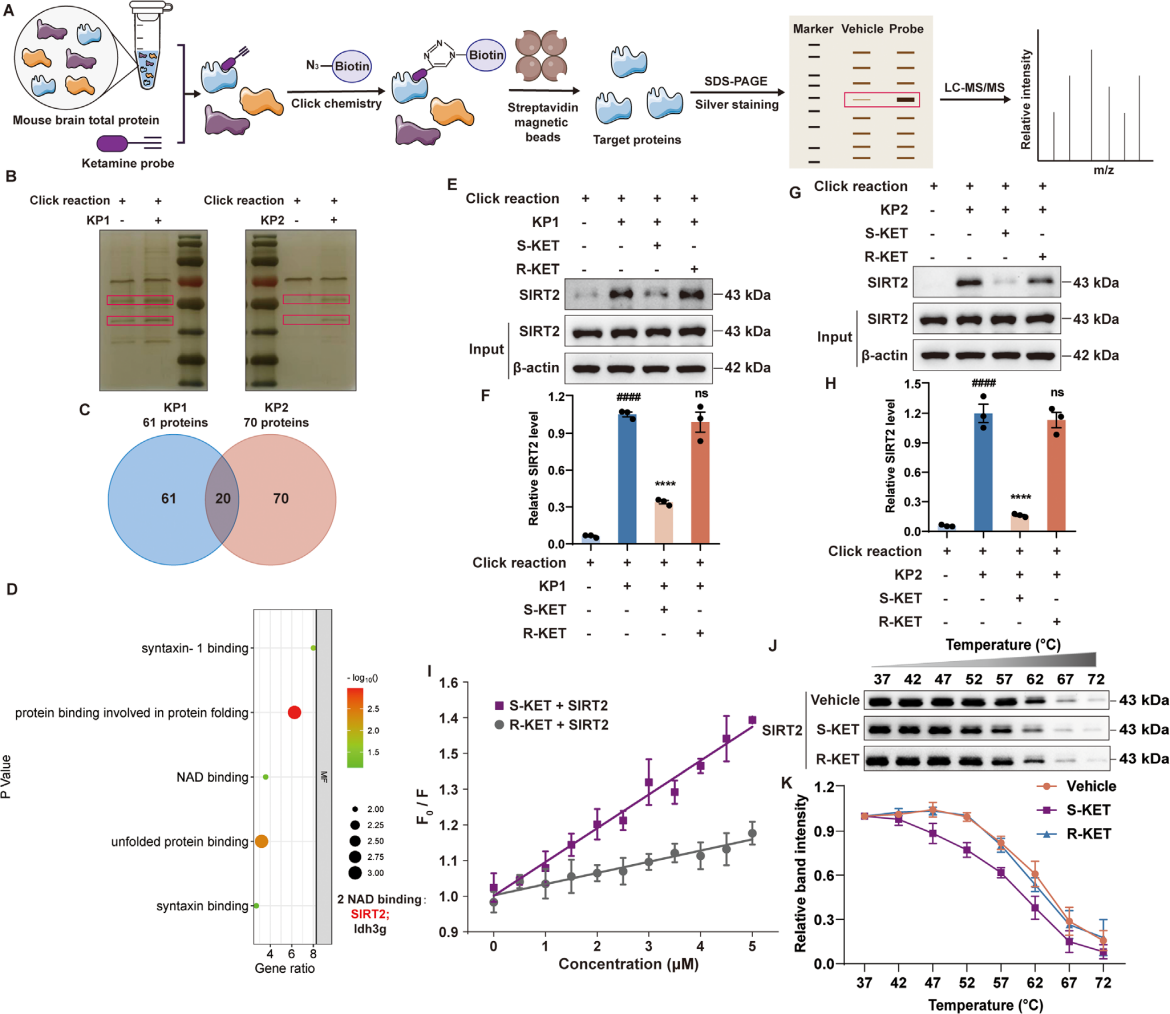
Target identification and verification of ketamine. A) Schematic illustration of the pull‐down assay for ketamine probes. B) Silver‐stained SDS‐PAGE showing proteins enriched by KP1 and KP2. C) Venn diagrams displaying the proteins identified by LC‐MS/MS, highlighting common proteins. D) Clustering of the 20 common proteins based on the molecular functions. E–H) Pull‐down and immunoblotting for target validation of SIRT2 with KP1 and KP2 (n = 3). I) Stern‐Volmer plot showing the intrinsic fluorescence titration of SIRT2 with S‐KET and R‐KET (n = 3). J) CETSA analysis to assess the interaction of endogenous SIRT2 with S‐KET and R‐KET (n = 3). K) Quantification of SIRT2 shown in panel (J). Data were presented as mean ± SEM and analyzed by one‐way ANOVA. # was used for comparisons with the vehicle group, * was used for comparisons with the KP1 or KP2 group. ####/****p < 0.0001. ns denoted *p* ≥ 0.05.

To identify the targets mediating the anti‐inflammatory effects of ketamine, two ketamine probes—KP1 and KP2—were designed and synthesized, with alkyne groups incorporated at different positions on the ketamine molecule (Scheme S1, Supporting Information). The protein target identification workflow is illustrated in **Figure** **2**A. KP1 and KP2 were incubated with mouse brain lysates, followed by attachment of biotin to the probes via a copper‐catalyzed click reaction. The proteins captured by KP1 and KP2 were isolated using streptavidin‐coated magnetic beads and analyzed by SDS‐PAGE with silver staining to reveal specific protein bands (Figure 2B). These bands were excised, proteolyzed into peptides, and analyzed using LC‐MS/MS. Proteins identified by KP1 and KP2 are listed in Dataset S1. KP1 and KP2 identified 61 and 70 potential targets, respectively, with 20 shared proteins (Figure 2C).

Further analysis of these 20 shared proteins using the DAVID database for molecular function revealed SIRT2 as the protein most closely associated with inflammation, warranting further verification (Figure 2D). Pull‐down assays confirmed that KP1 (Figure 2E,F) and KP2 (Figure 2G,H) directly bound to SIRT2, while S‐KET demonstrated competitive binding with the probes, whereas R‐KET did not (Figure 2E–H). To investigate the interaction between ketamine and SIRT2, the SIRT2 protein was prepared (Figure S1, Supporting Information), and binding titrations were conducted. S‐ketamine exhibited binding to SIRT2 with a dissociation constant of 4.7 ± 0.5 µM (Figure S2, Supporting Information). Notably, the Stern‐Volmer constant for S‐KET was significantly higher compared to R‐KET (Figure 2I), indicating a much stronger binding affinity of S‐KET for SIRT2.

To confirm the direct interaction between S‐KET and endogenous SIRT2, a cellular thermal shift assay (CETSA) was conducted. Results showed that S‐KET reduced the thermal stability of SIRT2, as indicated by a shift in its melting temperature, while R‐KET had no effect on SIRT2 stability (Figure 2J,K). Collectively, these results indicate that S‐KET directly binds to SIRT2, whereas R‐KET does not.

### 2.3 Q167 of SIRT2 is a Critical Residue for Interaction with S‐KET

**Figure 3:**
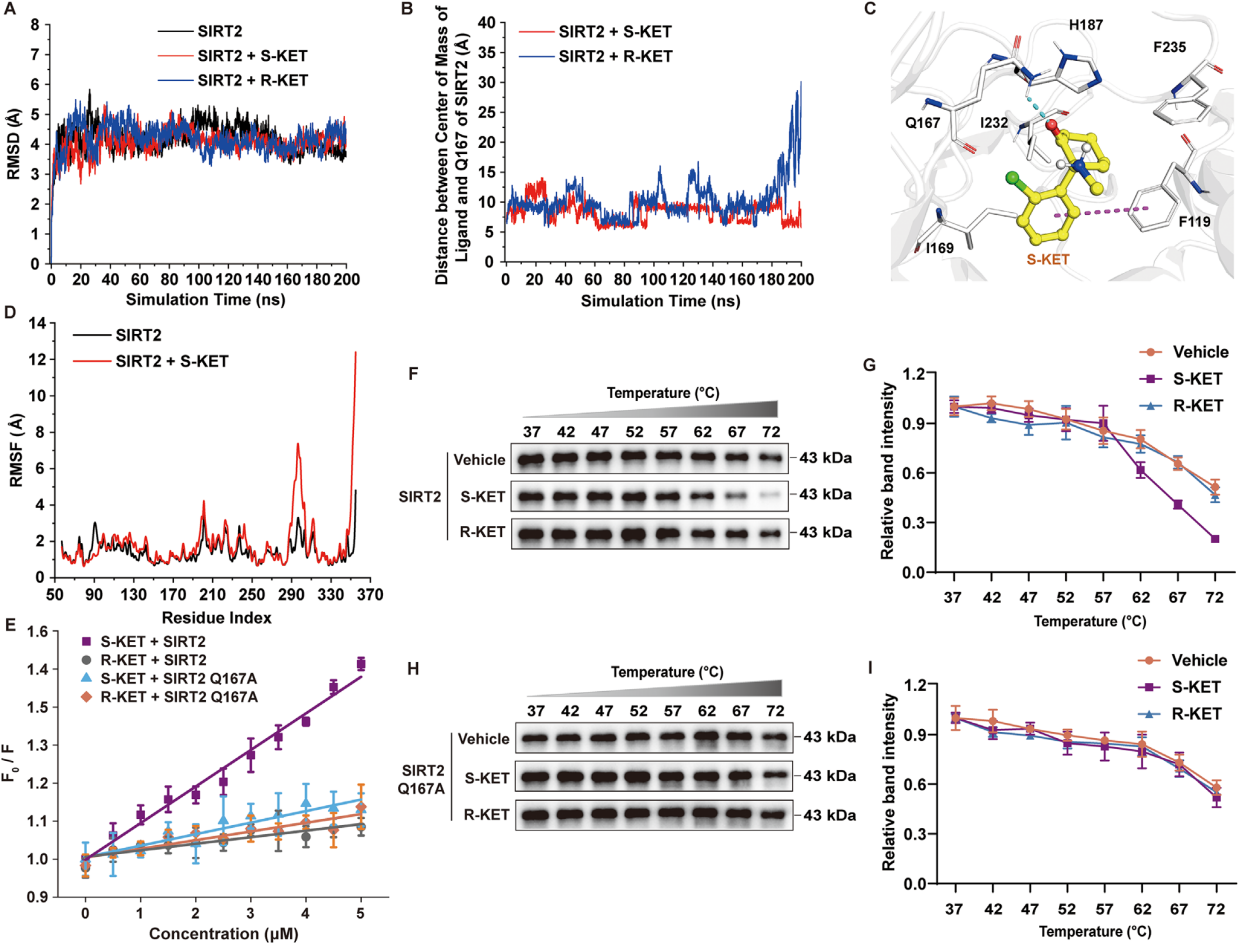
The mutant of SIRT2 Q167 disrupts the interaction between SIRT2 and S‐KET. A) RMSD values of protein backbone atoms for SIRT2 in ligand‐free SIRT2 (black), SIRT2/S‐KET (red), and SIRT2/R‐KET (blue) systems during 200 ns MD simulations. B) Changes in the distance between the center of mass of S‐KET (red) and R‐KET (blue) relative to Q167 of SIRT2 during 200 ns MD simulations. C) Interaction pattern of S‐KET binding to SIRT2, with key residues of SIRT2 and S‐KET shown as silver and yellow ball‐and‐stick, respectively. D) RMSF values of protein Cα atoms for SIRT2 in ligand‐free SIRT2 (black) and SIRT2/S‐KET (red) systems during last 40 ns MD simulations. E) Stern‐Volmer plot showing the intrinsic protein fluorescence titrations of SIRT2 and SIRT2 Q167A with S‐KET and R‐KET. F,H) Protein thermal shift assay of recombinant SIRT2 (F) and SIRT2 Q167A (H) proteins with S‐KET and R‐KET. G) Quantification of SIRT2 shown in panel (F). I) Quantification of SIRT2 Q167A shown in panel (H). All experiments were performed in triplicate, with data presented as mean ± SEM.

Molecular dynamics (MD) simulations were performed to investigate the interaction patterns of ketamine with SIRT2. The RMSD analysis of protein backbone atoms in ligand‐free SIRT2, S‐KET/SIRT2, and R‐KET/SIRT2 systems showed that all systems reached stability during the 200 ns simulations (**Figure** **3**A). Binding stability was assessed by monitoring the distance between each ligand and the center of mass of Q167, a residue near both S‐KET and R‐KET at the start of the simulations. S‐KET remained consistently close to Q167, while the distance between R‐KET and Q167 fluctuated significantly, leading to complete dissociation of R‐KET from SIRT2 at 180 ns (Figure 3B). Further analysis revealed that S‐KET formed π‐π stacking interactions with F119 and hydrophobic interactions with I169, H187, I232, and F235. Importantly, S‐KET also formed a hydrogen bond with Q167 (Figure 3C), an evolutionarily conserved residue between mice and humans whose mutation is known to reduce SIRT2's histone deacetylase activity.^[^ ^24^ ^]^ RMSF analysis showed that S‐KET binding increased the flexibility of certain regions of SIRT2 (Figure 3D), suggesting it could destabilize the SIRT2 structure, consistent with experimental CETSA data.

To validate the critical role of Q167, a recombinant SIRT2 Q167A mutant protein was produced (Figure S1, Supporting Information). The Stern‐Volmer constant for S‐KET binding to wild‐type SIRT2 was significantly higher than that for the SIRT2 Q167A mutant, indicating that the Q167A mutation substantially weakens the interaction between SIRT2 and S‐KET (Figure 3E). In contrast, no significant differences were observed in the interaction of R‐KET with either wild‐type SIRT2 or the SIRT2 Q167A mutant, and both interactions were significantly weaker compared to S‐KET binding to wild‐type SIRT2 (Figure 3E). Furthermore, thermal shift assays demonstrated that S‐KET reduced the thermal stability of wild‐type SIRT2, while R‐KET had no such effect (Figure 3F,G), consistent with previous CETSA data (Figure 2J,K). Neither S‐KET nor R‐KET altered the thermal stability of the SIRT2 Q167A mutant (Figure 3H,I). Collectively, these results indicate that Q167 of SIRT2 is essential for the interaction between SIRT2 and S‐KET.

### 2.4 S‐KET Enhances SIRT2 Interaction with p65 and Inhibits NF‐κB Activation

**Figure 4:**
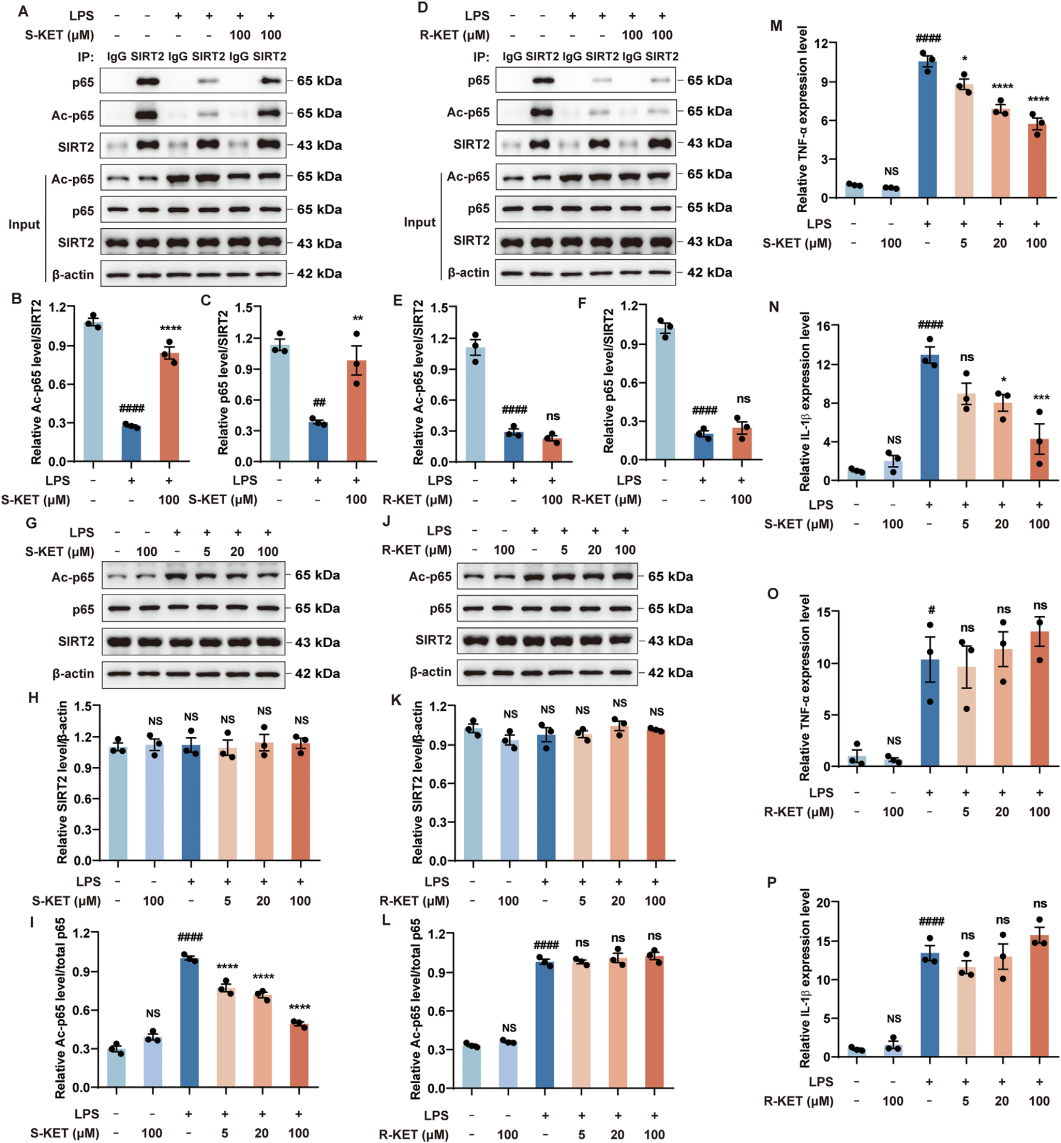
S‐KET improves the SIRT2‐p65 interaction, inhibits NF‐κB activity, and reduces LPS‐induced pro‐inflammatory factors overproduction. A–F) Co‐immunoprecipitation using an anti‐SIRT2 antibody, with p65 and Ac‐p65 detected by immunoblotting. G–L) The impact of S‐KET and R‐KET on SIRT2 (G, H, J, K) and LPS‐induced p65 acetylation (G, I, J, L). Total protein levels of p65 and β‐actin were used as references. M‐P) The effects of S‐KET and R‐KET on LPS‐induced mRNA expression of pro‐inflammatory factors TNF‐α (M, O) and IL‐1β (N, P) were assessed by qPCR. All experiments were performed in triplicate, with data presented as mean ± SEM and analyzed by one‐way ANOVA. # was used for comparisons with the vehicle group, * was used for comparisons with the LPS group. #/*p < 0.05, ##/**p < 0.01, ***p < 0.001, ####/****p < 0.0001. NS/ns denoted *p* ≥ 0.05. [Correction added on 16 May 2025, after first online publication: figure 4 caption and section title 2.4 is updated to accurately reflect the experimental findings and maintain the scientific integrity.]

SIRT2, a member of the Sirtuin family of NAD‐dependent deacetylases, plays a crucial role in regulating inflammation by deacetylating lysine 310 on the NF‐κB subunit p65, which results in reduced NF‐κB activation and suppression of downstream pro‐inflammatory gene expression, including TNF‐α and IL‐1β.^[^ ^25^, ^26^ ^]^ Co‐immunoprecipitation using an anti‐SIRT2 antibody found that S‐KET significantly enhanced the interaction between SIRT2 and both p65 and acetylated p65 (**Figure** **4**A–C), whereas R‐KET showed no effect at the same concentration (Figure 4D–F). LPS stimulation increased p65 acetylation levels (Figure 4G,I). While LPS, S‐KET, or the combination of LPS and S‐KET had no effect on protein expressions of SIRT2 (Figure 4G,H), treatment with S‐KET significantly inhibited LPS‐induced p65 acetylation in a dose‐dependent manner (Figure 4G,I). In contrast, R‐KET had no impact on LPS‐induced p65 acetylation (Figure 4J–L). These results indicate that S‐KET reduces acetylated p65 levels by enhancing the interaction between SIRT2 and acetylated p65, thereby inhibiting NF‐κB signaling activation, whereas R‐KET had no such effect.

To further assess the anti‐inflammatory potential of S‐KET and R‐KET, the expression levels of pro‐inflammatory cytokines TNF‐α (Figure 4M,O) and IL‐1β (Figure 4N,P) were measured. LPS stimulation increased the mRNA expression of these pro‐inflammatory factors, and S‐KET significantly reduced the LPS‐induced TNF‐α (Figure 4M) and IL‐1β (Figure 4N) mRNAs in a concentration‐dependent manner. In contrast, R‐KET at the same concentration did not inhibit the LPS induced overexpression of TNF‐α (Figure 4O) and IL‐1β (Figure 4P). These findings clearly indicate that S‐KET exerts a stronger anti‐inflammatory effect compared to R‐KET.

### 2.5 SIRT2 as a Crucial Target for the Anti‐Inflammatory Actions of S‐KET

**Figure 5:**
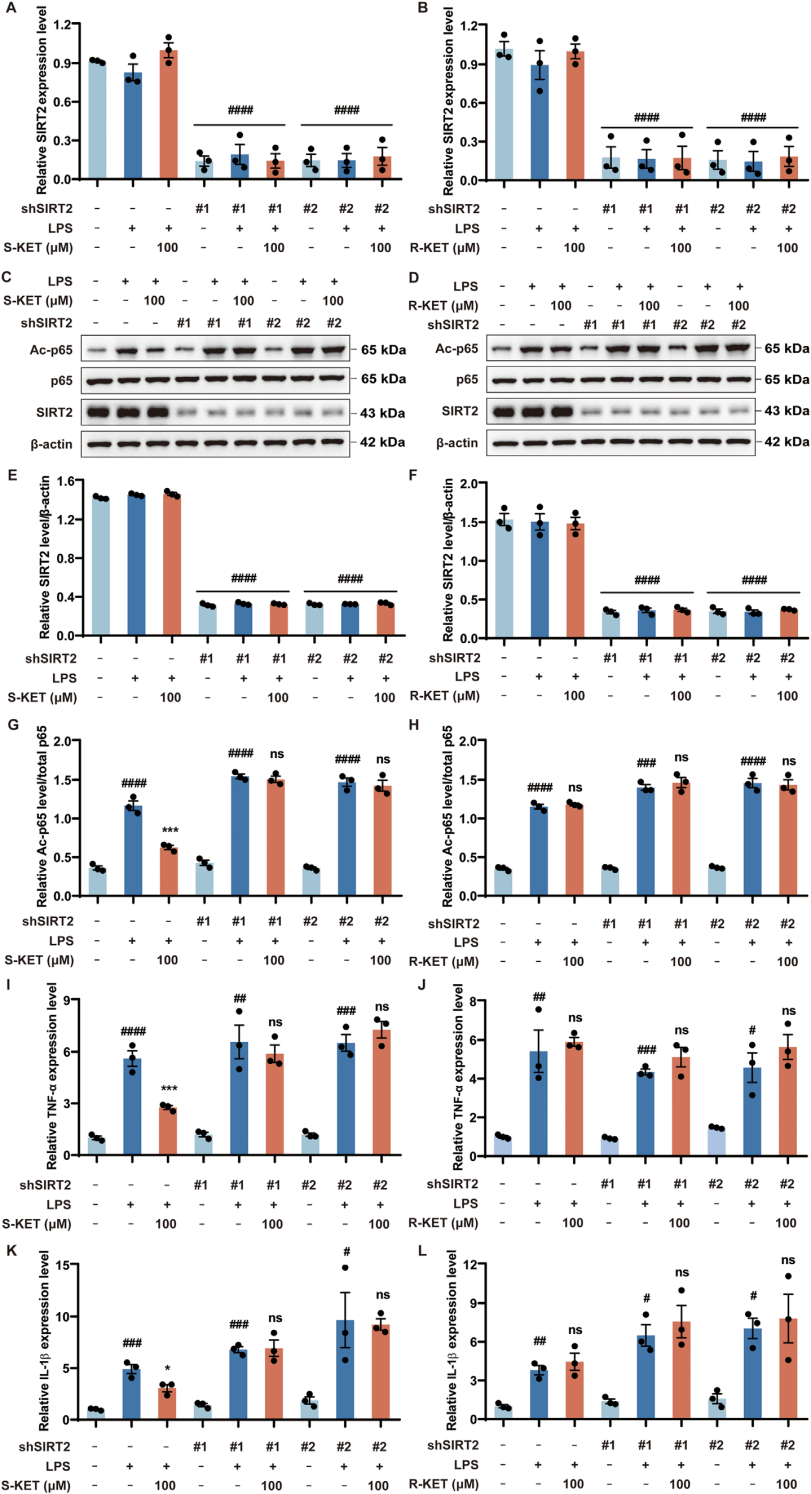
SIRT2 RNA interference diminishes the inhibitory effect of S‐KET on NF‐κB activity and LPS‐induced pro‐inflammatory factors. A,B) SIRT2 mRNA levels were measured in mock and SIRT2 knockdown BV‐2 cells treated with LPS, S‐KET, R‐KET, or the combination of LPS and KET. C–H) The effects of S‐KET and R‐KET on SIRT2 protein levels (C‐F) and LPS‐induced p65 acetylation (C, D, G, H) were assessed in mock BV‐2 cells and SIRT2 knockdown BV‐2 cells. Total protein levels of p65 and β‐actin were used as references. I–L) The impact of S‐KET and R‐KET on TNF‐α (I, J), and IL‐1β (K, L) mRNA expression were examined in mock BV‐2 cells and SIRT2 knockdown BV‐2 cells. All experiments were performed in triplicate, with data presented as mean ± SEM and analyzed by one‐way ANOVA. # was used for comparisons with the vehicle group, * was used for comparisons with the LPS group. #/*p < 0.05, ##*p* < 0.01, ###/***p < 0.001, ####/****p < 0.0001. ns denoted *p* ≥ 0.05.

To validate the role of SIRT2 in the anti‐inflammatory effects of S‐KET, a stable SIRT2 knockdown BV‐2 cell line was generated using shRNAs targeting SIRT2. The effects of S‐KET and R‐KET on SIRT2 mRNA and protein levels were first assessed in these knockdown cells. SIRT2 mRNA levels were reduced by over 80% in the SIRT2 knockdown cells and remained unaffected by treatment with LPS, S‐KET, R‐KET, or the combination of LPS and KET (**Figure** **5**A,B). Similarly, SIRT2 protein levels were reduced by over 70% in the SIRT2 knockdown cell line and also remained unchanged after treatment with LPS, S‐KET, R‐KET, or the combination of LPS and KET (Figure 5C–F). S‐KET effectively inhibited LPS‐induced p65 hyperacetylation in mock shRNA‐transfected cells (Figure 5C,G). However, S‐KET failed to inhibit LPS‐induced p65 hyperacetylation in SIRT2 knockdown cells, indicating that SIRT2 is necessary for S‐KET's anti‐inflammatory action (Figure 5C,G). In contrast, R‐KET did not alter LPS‐induced p65 acetylation in either mock shRNA‐transfected cells or SIRT2 knockdown cells (Figure 5D,H). Additionally, neither S‐KET nor R‐KET could suppress LPS‐induced overexpression of TNF‐α (Figure 5I,J) or IL‐1β (Figure 5K,L) mRNA levels in SIRT2 knockdown cells. These findings indicate that SIRT2 is crucial for the anti‐inflammatory effects of S‐KET.

**Figure 6:**
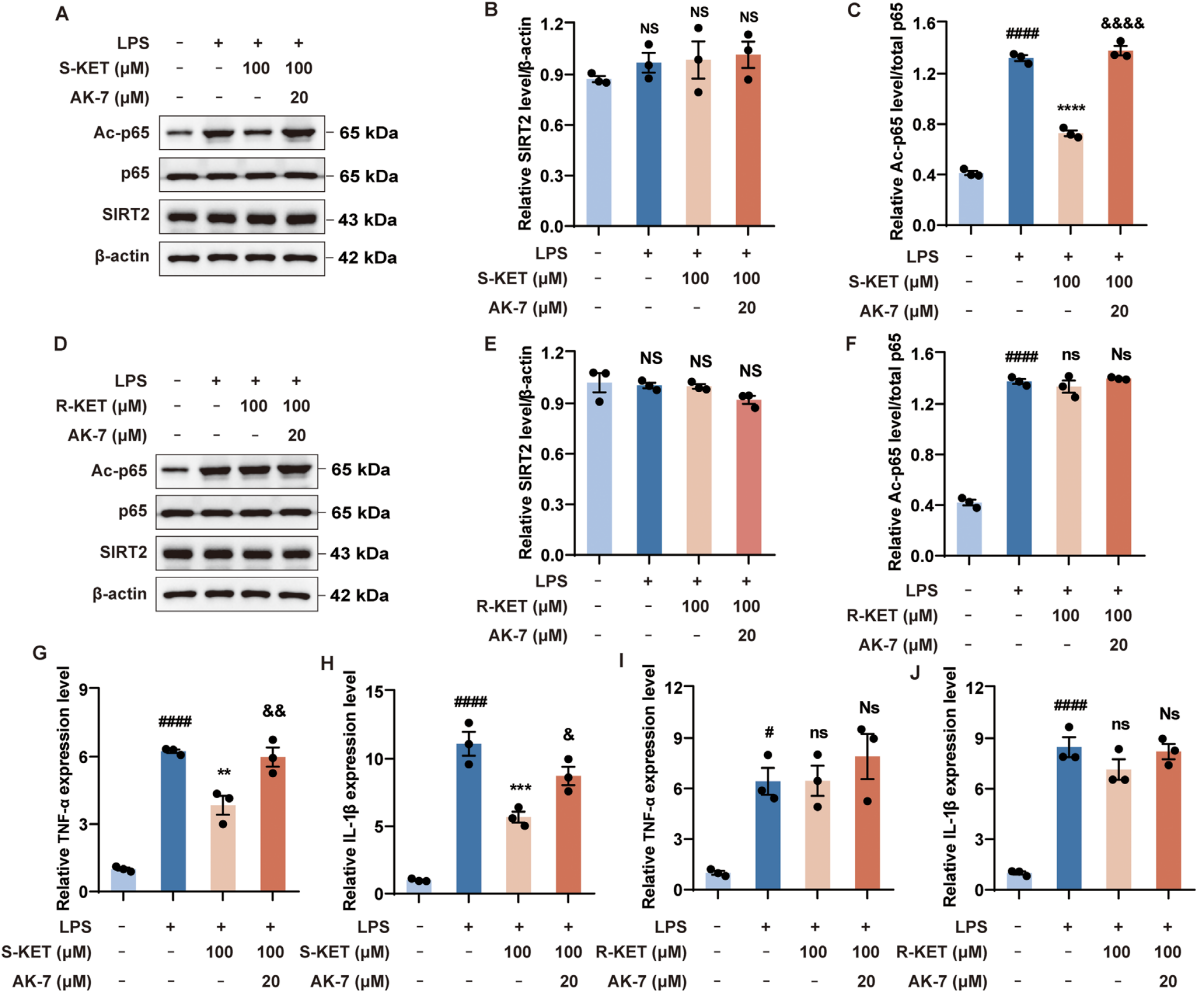
Pharmacological inhibition of SIRT2 attenuates S‐KET's ability to suppress NF‐κB signaling and LPS‐induced inflammatory factors. The effects of AK‐7 on the regulation of SIRT2 expression (A, B, D, E), p65 acetylation (A, C, D, F), and pro‐inflammatory factors TNF‐α (G, I) and IL‐1β (H, J) by S‐KET and R‐KET were analyzed in BV‐2 cells. Total p65 protein and β‐actin levels were used as references. All experiments were performed in triplicate, with data presented as mean ± SEM and analyzed by one‐way ANOVA. # was used for comparisons with the vehicle group, * was used for comparisons with the LPS group, & was used for comparisons with the LPS + S‐KET group. #/&*p* < 0.05, **/&&*p* < 0.01, ***p < 0.001, ####/****/&&&&*p* < 0.0001. NS/Ns/ns denoted *p* ≥ 0.05.

In addition to RNA interference, pharmacologic inhibition of SIRT2 by the selective inhibitor AK‐7 was also performed to further validate the role of SIRT2 in mediating the anti‐inflammatory effect of S‐KET. SIRT2 protein levels remained unaffected by treatment with LPS, S‐KET, R‐KET, or the combination of LPS and KET (**Figure** **6**A,B,D,E). AK‐7 significantly diminished the effectiveness of S‐KET in reducing LPS‐induced p65 acetylation (Figure 6A,C) as well as inhibiting LPS‐induced overexpression of TNF‐α (Figure 6G) and IL‐1β (Figure 6H) mRNAs. Co‐administration of AK‐7 with R‐KET has no effect on LPS induced p65 acetylation (Figure 6D,F) and TNF‐α (Figure 6I) and IL‐1β (Figure 6J) mRNAs overexpression.

Taken together, these results demonstrate that SIRT2 is a critical target for S‐KET in suppressing LPS‐induced pro‐inflammatory cytokine production by modulating p65 acetylation.

### 2.6 SIRT2 as a Key Target for the Antidepressant Effects of S‐KET In Vivo

**Figure 7:**
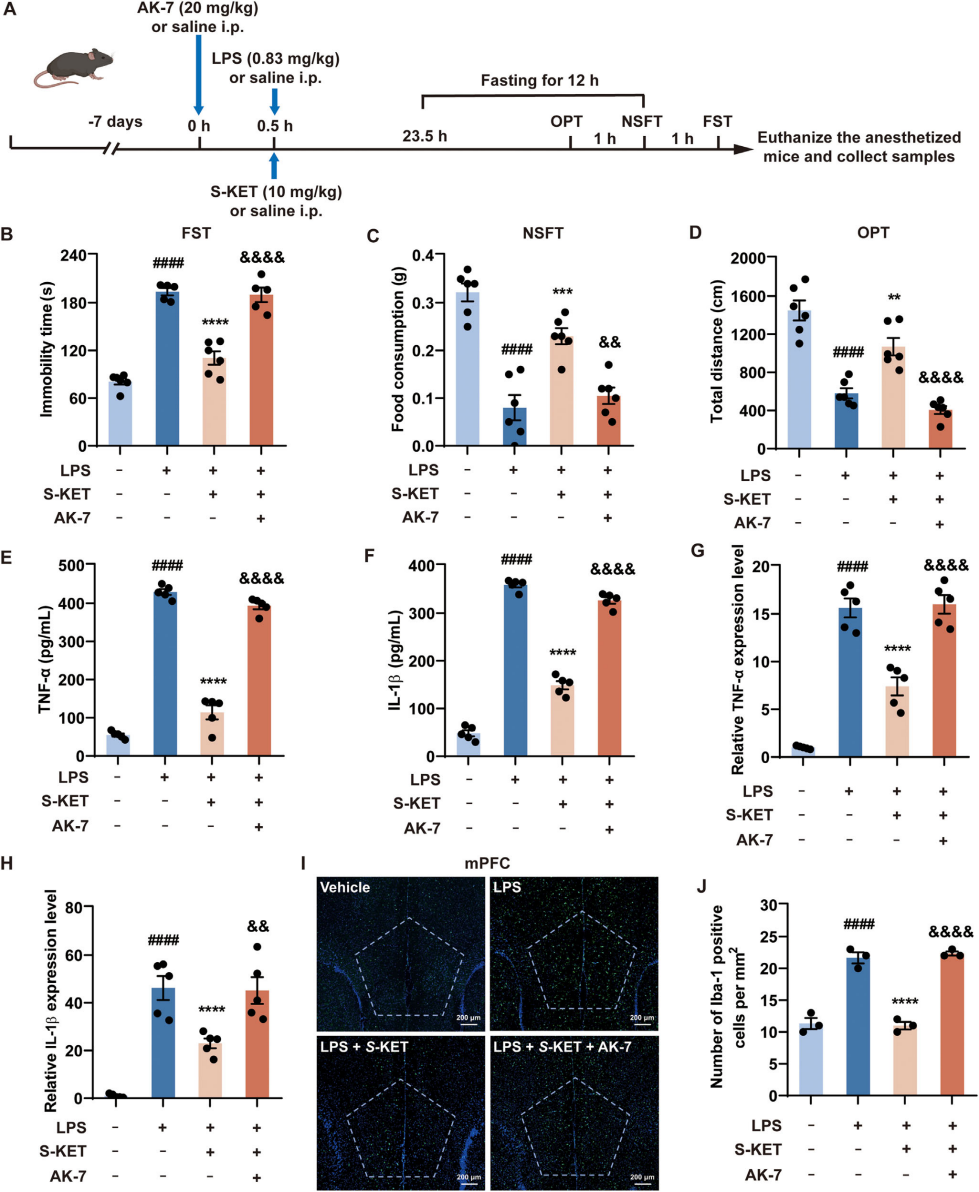
SIRT2 inhibition by AK‐7 reverses S‐KET's effects on LPS‐induced depressive‐like behaviors and inflammation. A) Schematic illustration of the experiment and timeline. B) Immobility time of FST (n = 5 – 6 per group). C) Food consumption of NSFT (n = 6 per group). D) Total distance of OPT (n = 6 per group). Serum samples and mPFC brain regions were collected following the behavioral tests. E,F) The impact of AK‐7 on S‐KET's effects on serum TNF‐α (E) and IL‐1β (F) was assessed by ELISA (n = 5). G,H) The effect of AK‐7 on S‐KET's modulation of TNF‐α (G) and IL‐1β (H) mRNA expression in the mPFC was measured by qPCR. (n = 5). I) Representative immunofluorescent images of the microglia activation marker Iba‐1 (n = 3). Scale bar: 200 µm. J) Quantification of Iba‐1^+^ microglial numbers shown in panel (I). Data were presented as mean ± SEM and analyzed by one‐way ANOVA. # was used for comparisons with the vehicle group, * was used for comparisons with the LPS group, & was used for comparisons with the LPS + S‐KET group. **/&&*p* < 0.01, ***p < 0.001, ####/****/&&&&*p* < 0.0001.

To determine whether S‐KET's alleviation of inflammation‐induced depressive‐like behavior in mice depends on SIRT2, an intraperitoneal injection of 20 mg kg^−1^ AK‐7, a selective SIRT2 inhibitor with good blood‐brain barrier permeability, was administered to specifically inhibit SIRT2 (**Figure** **7**A). LPS induced increased immobility time in the FST (Figure 7B), reduced food intake in the NSFT (Figure 7C), and decreased total distance traveled in the OFT (Figure 7D), all indicative of depressive‐like behavior. Treatment with S‐KET alleviated LPS‐induced depressive‐like behavior, whereas AK‐7 antagonized these therapeutic effects of S‐KET (Figure 7B–D).

Following the behavioral tests, the mice were sacrificed, and serum and mPFC samples were collected. ELISA was used to measure the serum levels of the pro‐inflammatory cytokines. Additionally, qPCR and immunofluorescence analyses were conducted to evaluate mRNA expression levels of TNF‐α and IL‐1β, as well as the expression of the microglial activation marker Iba‐1 in the mPFC. S‐KET significantly reduced LPS‐induced elevations in serum TNF‐α (Figure 7E) and IL‐1β levels (Figure 7F), as well as the increased mRNA expression levels of TNF‐α (Figure 7G) and IL‐1β (Figure 7H) and the expression of Iba‐1 in the mPFC (Figure 7I,J). However, these inhibitory effects were attenuated when SIRT2 was pharmacologically inhibited by AK‐7 (Figure 7E–J). These findings suggest that SIRT2 is a key target for the anti‐inflammatory and antidepressant effects of S‐KET in alleviating LPS‐induced depressive‐like behavior in vivo.

## 3 Discussion

Depression is a complex and multifaceted mental health disorder characterized by persistent feelings of sadness, hopelessness, and a lack of interest or pleasure in daily activities.^[^ ^1^, ^2^, ^3^ ^]^ The rapid‐onset antidepressant effects of S‐KET have been well‐documented in clinical settings. Its ability to alleviate depressive symptoms in MDD and TRD has been primarily linked to NMDA receptor antagonism, resulting in enhanced synaptogenesis and neuroplasticity.^[^ ^16^, ^17^, ^18^ ^]^ However, recent studies have highlighted the significant role of neuroinflammation in the pathophysiology of depression, with evidence pointing to the contribution of both peripheral and central inflammatory pathways to depressive symptoms.^[^ ^13^, ^14^, ^15^ ^]^ This study presents a comprehensive investigation of the differential effects of S‐KET and R‐KET on LPS‐induced depressive‐like behavior and neuroinflammation, demonstrating that S‐KET is more effective than R‐KET in ameliorating depressive symptoms in an LPS‐induced mouse model of depression. This finding underscores the significant impact of stereochemistry on ketamine's pharmacological actions, particularly in inflammation‐associated depression. Although several studies have reported anti‐inflammatory and antidepressant effects of R‐Ketamine (R‐KET), our findings appear to differ in this regard. It is worth noting that R‐KET is typically isolated from racemic ketamine, and variations in the purity of the isolated product could potentially influence experimental results. To ensure the accuracy of our study, both S‐KET and R‐KET were thoroughly characterized, achieving enantiomeric excess (*ee*) values of 100%, which minimizes the possibility of purity‐related interference.

Traditionally, most neuropsychiatric drugs have been thought to act by binding to extracellular targets on cell membranes—such as ion channels, receptors, and transport proteins, thereby activating classical signaling pathways and initiating intracellular cascades.^[^ ^27^ ^]^ However, recognizing that many small‐molecule drugs can cross cell membranes to enter cells, the potential roles of intracellular receptors as drug targets have recently garnered increased attention.^[^ ^28^ ^]^ In this study, the identification of SIRT2 as a key intracellular target of S‐KET's anti‐inflammatory actions offers a novel perspective on ketamine's mechanisms of action and provides an opportunity to develop selective SIRT2 modulators for the treatment of depression associated with neuroinflammation. Interestingly, R‐KET is unable to interact with SIRT2 and shows no apparent inhibition of LPS‐induced inflammation and depression‐like behaviors. This enantiomer‐specific effect underscores the complexity of ketamine's pharmacological profile and suggests that targeting SIRT2 could offer a novel strategy for developing more effective antidepressant therapies.

Inflammation‐induced depression is a well‐established model that reflects the pathological interplay between peripheral immune activation and central nervous system inflammation.^[^ ^21^, ^22^, ^23^ ^]^ The mPFC is a crucial brain region involved in emotion regulation, and its dysfunction is strongly associated with depression. Inflammatory responses, particularly the activation of microglia, can impair neural function, leading to disrupted emotion regulation. Furthermore, inflammation may alter the connectivity between the mPFC and other brain regions, such as the amygdala and hippocampus, by affecting neuroplasticity and synaptic pruning, which in turn exacerbates mood disorders.^[^ ^29^, ^30^, ^31^ ^]^ In this study, LPS treatment induced microglia activation and overexpression of the pro‐inflammatory cytokines TNF‐α and IL‐1β in the mPFC. Notably, S‐KET‐but not R‐KET‐was found to inhibit LPS‐induced microglia activation and inflammation in this region. These findings highlight the potential of S‐KET in modulating neuroinflammatory processes associated with depression. However, it is important to acknowledge the need for further exploration of how S‐KET modulates depression‐related neural circuits, particularly its interaction with SIRT2 and the roles of microglia and neurons in this process in future studies.

It is crucial to recognize that small molecules often engage in promiscuous interactions, allowing them to modulate multiple molecular targets simultaneously. S‐KET exemplifies this by interacting with a variety of targets, including NMDA and AMPA receptors, opioid receptors, and monoaminergic systems,^[^ ^32^, ^33^ ^]^ in addition to SIRT2. The ability of S‐KET to influence multiple pathways likely underpins its efficacy, highlighting its polypharmacological properties. Consequently, further investigation into these multifaceted interactions is essential to fully understand and optimize S‐KET's potential in treating complex neurological and psychiatric disorders.

In summary, this study provides strong evidence that S‐KET's antidepressant and anti‐neuroinflammatory effects are mediated through direct interaction with SIRT2, which modulates the NF‐κB pathway and influences p65 acetylation. Specifically, the Q167 residue of SIRT2 is essential for S‐KET binding, which ultimately suppresses NF‐κB activity and the overproduction of pro‐inflammatory factors. These findings significantly enhance our understanding of the molecular mechanisms underlying S‐KET's antidepressant actions and highlight SIRT2 as a promising therapeutic target for treating inflammation‐induced depression.

## 4 Experimental Section

### Materials

The details of the chemicals and solvents were provided in Table S1 (Supporting Information). Dulbecco's Modified Eagle Medium (DMEM) (C11995500BT), Phosphate‐Buffered Saline (PBS) (10010‐023), 0.25% Trypsin‐EDTA (25200‐072), Dynabeads M‐280 Streptavidin (11206D) beads, and Penicillin/Streptomycin (15140‐122) were obtained from Thermo Fisher Scientific. LPS (L2630) was sourced from Sigma‐Aldrich. Ultrapure LPS (tlrl‐3pelps), and puromycin (ant‐pr‐1) were purchased from InvivoGen. Protease inhibitor cocktails (20124ES10), Super ECL Detection Reagent (36208ES76), Trizol (10606ES60), Hifair III 1st Strand cDNA Synthesis SuperMix (11141ES), and SYBR Green (11201ES03) were obtained from YEASEN Biotech Co., Ltd. Phosphatase inhibitors (78420) were purchased from Roche. Fast Silver Stain Kit (P0017S), Mouse TNF‐α ELISA Kit (PT512), Mouse IL‐1β ELISA Kit (PI301), RIPA lysis buffer (P0013B), cell lysis buffer (P0013), and isopropyl‐β‐D‐thio‐galactoside (IPTG) (ST098) were obtained from Beyotime Biotechnology. Fast Site‐Directed Mutagenesis Kit (KM101) was sourced from TIANGEN BIOTECH Co., Ltd. Protein A/G Magnetic Beads (HY‐K0202), and AK‐7 (HY‐16691) were obtained from MedChemExpress. BCA Protein Assay Kit (MA0082) was purchased from Meilunbio Co., Ltd. High Affinity Ni‐NTA Resin (L00250) was obtained from Nanjing GenScript Biotech Co., Ltd. Fetal bovine serum (FBS) was sourced from PAN‐Seratech. S‐KET (86901445003581) was purchased from Jiangsu Hengrui Pharmaceuticals Co., Ltd. Racemic ketamine was provided by the Department of Public Security Jilin Province. R‐KET was resolved from racemic ketamine (Chiral resolution of racemic ketamine in Scheme S2, Supporting Information).The antibodies with the indicated dilutions were as follows: SIRT2 polyclonal antibody (Proteintech, 19655‐1‐AP, 1:200); SIRT2 monoclonal antibody (Proteintech, 66410‐1‐Ig, 1:3000); β‐actin (Proteintech, 66009‐1‐Ig, 1:10000); Rabbit IgG (Bioworld, BD0051, 1:1000); HRP‐conjugated Affinipure Goat Anti‐Mouse IgG (H+L) (Proteintech, SA00001‐1, 1:2000), HRP‐conjugated Affinipure Goat Anti‐Rabbit IgG (H+L) (Proteintech, SA00001‐2, 1:3000); Alexa Fluor 488 Goat Anti‐Mouse IgG H&L (Abcam, ab150113, 1:200); NF‐κB p65 (Cell Signaling Technology, 8242, 1:1000); Acetyl‐NF‐κB p65 (Lys310) (Cell Signaling Technology, 12629, 1:1000); Iba‐1 (Abcam, ab283319, 1:200); VeriBlot for IP Detection (Abcam, ab131366, 1:1000).

### Mice

C57BL/6J male mice (7‐8 weeks old, weighing 22–24 g) were sourced from Beijing Vital River Laboratory Animal Technology Co., Ltd. Mice were maintained in a standard environment with a temperature range of 22–24 °C, humidity levels of 40–60%, and a light cycle 12 h (8:00 AM to 8:00 PM). Mice had unlimited access to food and water and were given 7 days to adjust to the environment before the experiments began. All the animal experiments were approved by the Institutional Animal Care and Use Committee (IACUC).

### Drug Administration and Sample Collection

Mice were randomly assigned to four groups: the control group (intraperitoneal injection of 0.9% saline), the LPS group (0.83 mg kg^−1^), the LPS + S‐KET (10 mg kg^−1^) group, and the LPS + R‐KET (10 mg kg^−1^) group. LPS and ketamine were administered simultaneously via intraperitoneal injection. Fasting began 12 h after treatment, with the open field test (OFT) taking place after 11 h of fasting. The novelty‐suppressed feeding test (NSFT) was carried out 12 h into the fasting period, and the forced swimming test (FST) was performed 1 h after completing the NSFT. After the behavioral test, mice were anesthetized with isoflurane and euthanized. The brain was then dissected, and the mPFC region was isolated.

### FST

A 30 cm high, 20 cm diameter glass cylinder is filled with 15 cm of water at 23 ± 1 °C, ensuring that each mouse's tail does not reach the bottom. Each mouse is carefully placed in the water, and its swimming behavior is recorded with a camera. After 6 min, the mouse is removed, and the software calculates the total immobility time during the final 4 min of the test (defined as the time with only minimal movement needed to keep the head above water). The water in the cylinder is replaced after each trial.

### NSFT

Mice were fasted for 12 h before the experiment, with free access to water. During testing, each mouse was positioned facing the wall in a 40 cm × 40 cm × 40 cm open white box, with two pre‐weighed food pellets placed at the center. A camera recorded the mouse's activity over 15 min, after which the food pellets were reweighed to determine the amount consumed. After each trial, the box was cleaned with 75% alcohol to prevent any environmental interference in future tests.

### OFT

Each mouse is individually placed in the center of a 40 cm × 40 cm × 40 cm open white experimental box, with a 20 cm × 20 cm area defined as the central zone. A camera records the mouse's movements over a 5 min free exploration period, and software calculates the total distance traveled. After each trial, the box is cleaned with 75% alcohol to prevent environmental contamination in the following tests.

### Cell Culture

The mouse microglial cell line BV‐2 was obtained from the China Center for Type Culture Collection (CCTCC) (RRID: CVCL_0182). The human embryonic kidney epithelial cell line HEK‐293T was sourced from the American Type Culture Collection (ATCC) (RRID: CVCL_0063). Both BV‐2 and HEK‐293T cells were cultured in DMEM supplemented with 10% FBS, 50 units per mL penicillin, and 50 µg mL^−1^ streptomycin at 37 °C in a 5% CO_2_ incubator. Prior to experiments, cells were tested for mycoplasma to ensure the absence of contamination.

### qPCR

BV‐2 cells were plated at a density of 2 × 10^5^ cells mL^−1^ in 6‐well plates. After overnight incubation, the medium was replaced with DMEM, and different concentrations of S‐KET or R‐KET were added for 2 h before adding LPS (20 ng mL^−1^). After 24 h, total RNA was extracted using Trizol according to the manufacturer's instructions. The Hifair III 1st Strand cDNA Synthesis SuperMix for qPCR was used to synthesize cDNA following the manufacturer's instructions. qPCR was conducted on a TOptical Real‐Time qPCR Thermal Cycler (Analytik Jena, Thuringia, GER) using the SYBR Green method, with Rpl27 as the reference gene. Data analysis was performed using the ^ΔΔ^Ct method. Primers of TNF‐α, IL‐1β, and Rpl27 were obtained from Sangon Biotech Co., Ltd., with primer sequences were provided in Table S2 (Supporting Information).

For qPCR of brain tissue, mice were deeply anesthetized and perfused with saline. The brain was then extracted, and the mPFC region was dissected. The tissues were promptly placed in liquid nitrogen. Following homogenization in Trizol, total RNA was isolated from the mPFC. The RNA was reverse‐transcribed into cDNA, and qPCR was performed as described above.

### ELISA

Whole blood from the mice was left at room temperature for 2 h to facilitate natural clotting and serum separation. The sample was then centrifuged at 4 °C at 2000 rpm for 10 min to obtain the yellow supernatant as the mouse serum sample. TNF‐α and IL‐1β levels were assessed using mouse ELISA kits following the manufacturer's instructions.

### Immunofluorescence Staining

The mPFC region was fixed in 4% paraformaldehyde for 24 h and subsequently embedded in paraffin. Coronal brain sections measuring 5 µm thick were then prepared using a vibratome. The sections were washed three times with PBS for 5 min each. The sections were incubated in a 3% hydrogen peroxide solution at room temperature in the dark for 25 min, followed by three additional washes with PBS. After incubation with 10% goat serum at room temperature for 30 min, the sections were incubated overnight at 4 °C with primary antibodies. Following three washes with PBS, the sections were incubated with secondary antibodies in the dark for 50 min. The sections were incubated with tyrosine salt fluorescent dye at room temperature for 20 min, followed by three washes with PBS for 5 min each. The sections were then incubated with DAPI staining solution in the dark for 10 min and washed three times with PBS for 5 min each. Finally, the sections were examined using confocal microscopy, and fluorescence intensity was analyzed with QuPath software.

### Pull‐Down Assay for Identifying the Target Protein

C57BL/6J mice were anesthetized, and the brain tissue was quickly frozen in liquid nitrogen. The tissue was homogenized in a pre‐cooled mortar with 1 mL of lysis buffer (25 mm HEPES pH 8.0, 150 mm KCl, 5 mm EDTA, 0.5% v/v NP‐40, protease inhibitors, and PMSF) at 4 °C. The homogenate was transferred to a 1.5 mL centrifuge tube, sonicated (150 W for 3 s with 5‐s pauses for a total of 2 min), and centrifuged at 4 °C at 12000 rpm for 15 min to collect the supernatant. Protein concentration was assessed using a BCA protein assay kit and adjusted to 1 mg mL^−1^ with lysis buffer. The KP1 or KP2 (10 µm) was added and an equal volume of DMSO (1/1000, v/v) as a vehicle, and the mixture was incubated at room temperature for 2 h. The click reaction was performed using 1 mm CuSO_4_, 100 µm TBTA, 1 mm TCEP, 80 µm Biotin‐N_3_, and 5% t‐BuOH, followed by a 1 h incubation. Proteins were enriched using 20 µL of streptavidin magnetic beads, which were washed three times with PBS and incubated at room temperature for 1.5 h. After three additional washes with PBS containing 0.1% Tween‐20, 40 µL of 2× loading buffer was added, mixed, and heated at 100 °C for 10 min. SDS‐PAGE gel electrophoresis was performed, and the separation gel was cut for silver staining following the rapid silver staining kit protocol. The differential bands obtained from silver staining were cut out, and the proteins were identified using LC‐MS/MS.

### Pull‐Down Assay to Verify the Interaction Between Ketamine and SIRT2

BV‐2 cells were harvested upon reaching over 90% confluence. Following two washes with PBS, the cells were lysed using a buffer containing 25 mm HEPES (pH 8.0), 150 mm KCl, 5 mm EDTA, 0.5% NP‐40, 1% protease inhibitor cocktail, and PMSF. The lysates were centrifuged at 4 °C for 10 min at 12000 rpm, and the protein concentration was adjusted to 1 mg mL^−1^ using the BCA assay kit. The KP1 or KP2 (10 µm) was added and an equal volume of DMSO (1/1000, v/v) as a vehicle, either alone or in combination with 50 µm S‐KET or R‐KET, and incubated at room temperature for 2 h. The subsequent procedure is consistent with the pull‐down assay for identifying the target protein. The protein samples were detected by immunoblotting with the corresponding primary antibodies.

### Immunoblotting

The protein samples were separated by SDS‐PAGE and subsequently transferred to PVDF membranes. The membranes were blocked with 5% nonfat dry milk for 1 h, then incubated overnight at 4 °C with the specific primary antibody. After the primary antibody incubation, the membranes were washed three times with Tris‐buffered saline containing 0.1% Tween 20 (TBST) for 5 min each time. The membranes were then incubated at room temperature for 1.5 h with a secondary HRP‐conjugated antibody. After washing three times with TBST, proteins were detected using the Super ECL Detection Reagent and imaged with the Tanon‐5200 Multi system. Densitometric analysis was carried out using ImageJ.

### CETSA

BV‐2 cells were seeded at a density of 2 × 10^5^ cells mL^−1^ in a 10 cm dish. After 24 h of incubation, the medium was removed, and cells were washed twice with cold PBS. Cells were harvested by scraping and collected by centrifugation at 2000 rpm for 3 min at 4 °C. The cell pellet was then resuspended in RIPA lysis buffer with 1% protease inhibitor cocktail and lysed on ice for 15 min. Lysates were centrifuged at 12000 rpm for 15 min at 4 °C to collect the supernatants, which were subsequently incubated with either 1 mM S‐KET, 1 mM R‐KET, or a vehicle control (sterile water) for 2 h at room temperature. Each supernatant was then divided into 60 µL aliquots in microtubes, heated to various temperatures (37, 42, 47, 52, 57, 62, 67, and 72 °C) for 5 min using a Long Gene A200 thermal cycler, and then cooled to 25 °C for 3 min. Samples were centrifuged at 15000 rpm for 20 min at 4 °C to separate precipitates. The supernatants were denatured in 2× loading buffer at 100 °C for 10 min and analyzed by immunoblotting with the SIRT2 antibody.

### Protein Expression and Purification

The bacterial expression vector pET28a‐His‐tag‐SIRT2 was created by inserting a PCR amplification fragment of mouse SIRT2. Additionally, the bacterial expression vector pET28a‐His‐tag‐SIRT2, with a mutation from glutamine 167 to alanine (Q167A), was generated from pET28a‐His‐tag‐SIRT2 using the Fast Site‐Directed Mutagenesis Kit according to the manufacturer's instructions. His‐tagged SIRT2 and His‐tagged SIRT2 Q167A proteins were expressed in Rosetta with 1 mm IPTG for 4 h at 30 °C, followed by purification on High Affinity Ni‐Charged Resin according to the manufacturer's instructions.

### Fluorescence Titration

Fluorescence titrations for SIRT2 (0.5 µm) and SIRT2 Q167A (0.5 µm) with ketamine were performed on a Cary Eclipse spectrofluorometer at room temperature, using a 2 × 10 mm quartz cell. The excitation wavelength was set to 280 nm to excite the Tyr and Trp residues, and emission was recorded from 310 to 450 nm. Fluorescence intensity at 337 nm for SIRT2 or SIRT2 Q167A was plotted against the concentrations of S‐KET or R‐KET.

### Molecular Docking

The crystal structure of the SIRT2 protein was obtained from the human SIRT2‐ADPR dimer complex (PDB ID: 5D7O).^[^ ^34^ ^]^ Missing hydrogen atoms were added using Maestro at pH = 7.0.^[^ ^35^ ^]^ S‐KET and R‐KET were docked to the SIRT2 protein using Autodock Vina 1.1.2,^[^ ^36^ ^]^ with a docking box size of 58 Å × 50 Å × 40 Å to encompass the entire protein (Figure S3, Supporting Information). Five docking conformations were generated, and the pose with the highest binding affinity to SIRT2 was selected to build simulation systems for subsequent molecular dynamics simulations (Figure S3, Supporting Information).

### Molecular Dynamics Simulation

Simulation systems were constructed using the CHARMM‐GUI web server.^[^ ^37^ ^]^ The ligand‐free SIRT2, SIRT2/S‐KET, and SIRT2/R‐KET complexes were each positioned at the center of a 95 Å × 95 Å × 95 Å box, with TIP3P water molecules and 0.15 mol L^−1^ NaCl added to simulate a physiological environment. All molecular dynamics (MD) simulations were conducted using GROMACS 2021^[^ ^38^ ^]^ with periodic boundary conditions. The CHARMM36 m force field^[^ ^39^ ^]^ was applied for proteins, TIP3P water, and ions, while force field parameters for protonated S‐KET and R‐KET were obtained using Antechamber.^[^ ^40^ ^]^ The LINCS algorithm^[^ ^41^ ^]^ was used to constrain all bonds involving hydrogen atoms. Van der Waals interactions were switched at 10 Å and cut off at 12 Å. Long‐range electrostatic interactions were managed by the particle‐mesh Ewald (PME)^[^ ^42^ ^]^ method with a cut‐off of 12 Å. Each system underwent initial energy minimization using the steepest descent algorithm, followed by equilibration for 0.125 ns. Harmonic constraints were applied to the protein heavy atoms at the start of equilibration, with force constants of 0.956 kcal/(mol·Å^2^) for the backbone and 0.0956 kcal/(mol·Å^2^) for side chains. After equilibration, each system was subjected to 200 ns of unconstrained MD simulation under the isothermal‐isobaric ensemble (NPaT). Pressure and temperature were maintained at 1 atm and 310.15 K using the Parrinello‐Rahman and Nosé‐Hoover coupling algorithms, respectively.^[^ ^43^, ^44^, ^45^, ^46^ ^]^ Root‐mean‐square deviations (RMSDs) and root‐mean‐square fluctuations (RMSFs) were calculated using internal GROMACS modules.^[^ ^38^ ^]^ Protein‐ligand interactions were analyzed with MDAnalysis 2.7^[^ ^47^ ^]^ and ProLIF 2.0,^[^ ^48^ ^]^ while simulation trajectories were visualized using VMD 1.9.4^[^ ^49^ ^]^ and PyMol 2.6.0.^[^ ^50^ ^]^

### Protein Thermal Shift

The protein thermal shift assay was carried out using 0.1 µM of purified SIRT2 or SIRT2 Q167A protein following the same procedure as described for CESTA above.

### Signaling Pathway Sample Preparation

BV‐2 cells were plated at a density of 5 × 10^5^ cells per well in a 6‐well plate. After incubating for 24 h, the medium was replaced with DMEM, and various concentrations of S‐KET or R‐KET were added for 2 h before the addition of 20 ng mL^−1^ of LPS. After stimulation with LPS for 1 h, cells were washed twice with ice‐cold PBS and lysed in a cell lysis buffer containing protease and phosphatase inhibitors on ice for 30 min. The supernatant was obtained by centrifuging at 12000 rpm for 10 min at 4 °C, and the protein concentration was determined using a BCA kit. The sample was then boiled with 2× loading buffer at 100 °C for 10 min for immunoblotting.

### Co‐Immunoprecipitation (Co‐IP)

BV‐2 cells were seeded at a density of 1 × 10^7^ cells in 100 mm culture dishes. After incubating for 24 h, the medium was replaced with DMEM, and either S‐KET (100 µm) or R‐KET (100 µm) was added for 2 h before introducing LPS (20 ng mL^−1^) for 1 h. The cells were then washed twice with ice‐cold PBS and lysed in 1 mL of Co‐IP lysis buffer (25 mm Tris pH 8.0, 150 mm KCl, 5 mm EDTA, 0.5% NP‐40) supplemented with a complete protease inhibitor cocktail, 1 mm DTT, and 1 mm PMSF, and incubated on ice for 30 min. The supernatant was obtained by centrifuging at 12000 rpm for 12 min at 4 °C. Either the anti‐SIRT2 antibody or rabbit anti‐IgG antibody was added, and the samples were gently rotated on a shaker at 4 °C for overnight incubation. 40 µL of washed magnetic beads were then added, and the samples were gently rotated and incubated on a shaker at room temperature for 1.5 h. The magnetic beads were washed three times with PBS and boiled with 80 µL of 2 × loading buffer at 100 °C for 10 min for immunoblotting.

### Construction of the Stable BV‐2 Cell Line with SIRT2 Interference

The primers for mock and SIRT2 short hairpin RNAs were annealed through gradient cooling and subsequently cloned into the pLKO.1‐puro vector. HEK‐293T cells were co‐transfected with 10 µg of the interference plasmid, 6.5 µg of the packaging plasmid pMDlg/pRRE, 2.5 µg of pRSV‐Rev, 3.5 µg of VSV‐G, and 10 µg of branched polyethyleneimine to generate lentivirus. After 48 h, the lentivirus supernatant was filtered through a 0.45 µm filter and added to BV‐2 cells at a density of 5 × 10^5^ cells per well in 6‐well plates, followed by a 24 h incubation. After the infection period, the medium was replaced with DMEM supplemented with 10% FBS, 50 units per mL penicillin, and 50 µg mL^−1^ streptomycin. After 48 h, cells were screened with 2 µg mL^−1^ puromycin for each passage. Following two weeks of screening, polyclonal cell lines underwent single‐cell isolation using the gradient dilution method. The expression levels of SIRT2 mRNA and protein in monoclonal cell lines were assessed by qPCR and immunoblotting to identify and select the optimal cell line. SIRT2, TNF‐α, and IL‐1β were detected by qPCR, and signaling pathway analysis was performed via immunoblotting in the monoclonal cell line, as described above. Primers of mock and SIRT2 short hairpin RNAs were obtained from Sangon Biotech Co., Ltd., with sequences provided in Table S3 (Supporting Information).

### In Vitro Pharmacological Experiments

BV‐2 cells were plated in a 6‐well plate. After 24 h, the medium was changed to DMEM, and 20 µm of AK‐7 was added, along with 100 µm of either S‐KET or R‐KET. Following a 2 h incubation, the cells were stimulated with 20 ng mL^−1^ of LPS, and stimulation times were adjusted based on the experimental design. TNF‐α and IL‐1β were detected using qPCR, and signaling pathway analysis was carried out by immunoblotting, as described above.

### In Vivo Pharmacological Experiments

Mice were randomly divided into four groups: the control group (intraperitoneal injection of 0.9% saline), the LPS group (0.83 mg kg^−1^), the LPS + S‐KET (10 mg kg^−1^) group, and the LPS + S‐KET + AK‐7 (20 mg kg^−1^) group. 0.5 h after the intraperitoneal injection of 20 mg kg^−1^ of AK‐7, LPS, and S‐KET were administered intraperitoneally at the same time. Sample collection, ELISA, immunofluorescence staining, and qPCR were performed as described above.

### Statistical Analysis

Data were presented as the mean ± SEM, and one‐way analysis of variance (ANOVA) was conducted for statistical analysis. All analyses were performed using GraphPad Prism 8.0. Statistical significance was indicated above the bars in each figure, with *p* < 0.05 considered statistically significant for all analyses.

## Conflict of Interest

The authors declare no conflict of interest.

## Author Contributions

C.L. and X.Z. contributed equally to this work. X. W. designed the research; C. L., X. Z., M. L., C. Z., H. Z., and H. W. performed the experiments; C. L., X. Z., C. Z., H. Z., and H. W. analyzed the data; C. L., X. Z., and H. W. wrote the manuscript; H.L. and X. W. edited the manuscript. All authors read and approved of the final manuscript.

## Supporting information



Supporting Information

Supporting Information

## Data Availability

The data that support the findings of this study are available from the corresponding author upon reasonable request.

## References

[1] Y. Chai , Y. I. Sheline , D. J. Oathes , N. L. Balderston , H. Rao , M. Yu , Trends Cogn. Sci. 2023, 27, 814. 37286432 10.1016/j.tics.2023.05.006 PMC10476530

[2] S. M. Gold , O. Kohler‐Forsberg , R. Moss‐Morris , A. Mehnert , J. J. Miranda , M. Bullinger , A. Steptoe , M. A. Whooley , C. Otte , Nat. Rev. Dis. Primers 2020, 6, 69. 32820163 10.1038/s41572-020-0200-2

[3] S. Marwaha , E. Palmer , T. Suppes , E. Cons , A. H. Young , R. Upthegrove , Lancet 2023, 401, 141. 36535295 10.1016/S0140-6736(22)02080-3

[4] L. Cui , S. Li , S. Wang , X. Wu , Y. Liu , W. Yu , Y. Wang , Y. Tang , M. Xia , B. Li , Signal Transduct. Target. Ther. 2024, 9, 30. 38331979 10.1038/s41392-024-01738-y PMC10853571

[5] I. V. Mousten , N. V. Sorensen , R. H. B. Christensen , M. E. Benros , JAMA Psychiatry 2022, 79, 571. 35442429 10.1001/jamapsychiatry.2022.0645 PMC9021989

[6] Y. Zhou , C. Wang , X. Lan , H. Li , Z. Chao , Y. Ning , J. Neuroinflammat. 2021, 18, 200. 10.1186/s12974-021-02245-5 PMC8444441 34526064

[7] D. R. Goldsmith , M. Bekhbat , N. D. Mehta , J. C. Felger , Biol. Psychiatry 2023, 93, 405. 36725140 10.1016/j.biopsych.2022.11.003 PMC9895884

[8] E. F. Osimo , T. Pillinger , I. M. Rodriguez , G. M. Khandaker , C. M. Pariante , O. D. Howes , Brain Behav. Immun. 2020, 87, 901. 32113908 10.1016/j.bbi.2020.02.010 PMC7327519

[9] S. Poletti , M. G. Mazza , F. Benedetti , Transl. Psychiatry 2024, 14, 247. 38851764 10.1038/s41398-024-02921-z PMC11162479

[10] Y. Pan , J. Chen , Y. Zhang , Y. Ren , Z. Wu , Q. Xue , S. Zeng , C. Fang , H. Zhang , L. Zhang , C. Liu , J. Zeng , Mol. Pharmaceut. 2024, 21, 1804. 10.1021/acs.molpharmaceut.3c01115 38466359

[11] H. Wang , Y. He , Z. Sun , S. Ren , M. Liu , G. Wang , J. Yang , J. Neuroinflammat. 2022, 19, 132. 10.1186/s12974-022-02492-0 PMC9168645 35668399

[12] B. Li , W. Yang , T. Ge , Y. Wang , R. Cui , Pharmacol. Res. 2022, 179, 106145. 35219870 10.1016/j.phrs.2022.106145

[13] B. Channer , S. M. Matt , E. A. Nickoloff‐Bybel , V. Pappa , Y. Agarwal , J. Wickman , P. J. Gaskill , Pharmacol. Rev. 2023, 75, 62. 36757901 10.1124/pharmrev.122.000618 PMC9832385

[14] C. Chu , D. Artis , I. M. Chiu , Immunity 2020, 52, 464. 32187517 10.1016/j.immuni.2020.02.017 PMC10710744

[15] O. Le Thuc , C. Garcia‐Caceres , Nat. Metab. 2024, 6, 1237. 38997442 10.1038/s42255-024-01079-8

[16] E. D. Ballard , C. A. Zarate Jr. , Nat. Commun. 2020, 11, 6431. 33353946 10.1038/s41467-020-20190-4 PMC7755908

[17] J. W. Kim , K. Suzuki , E. T. Kavalali , L. M. Monteggia , Trends Mol. Med. 2023, 29, 364. 36907686 10.1016/j.molmed.2023.02.003 PMC10101916

[18] S. Y. Smith‐Apeldoorn , J. K. Veraart , J. Spijker , J. Kamphuis , R. A. Schoevers , Lancet Psychiatry 2022, 9, 907. 36244360 10.1016/S2215-0366(22)00317-0

[19] P. Zanos , R. Moaddel , P. J. Morris , L. M. Riggs , J. N. Highland , P. Georgiou , E. F. R. Pereira , E. X. Albuquerque , C. J. Thomas , C. A. Zarate Jr. , T. D. Gould , Pharmacol. Rev. 2018, 70, 621. 29945898 10.1124/pr.117.015198 PMC6020109

[20] J. N. Johnston , I. D. Henter , C. A. Zarate Jr. , Pharmacol. Ther. 2023, 246, 108431. 37146727 10.1016/j.pharmthera.2023.108431 PMC10213151

[21] Y. Wang , C. Gao , T. Gao , L. Zhao , S. Zhu , L. Guo , Brain Behav. Immun. 2021, 94, 225. 33607235 10.1016/j.bbi.2021.02.004

[22] F. R. Xu , Z. H. Wei , X. X. Xu , X. G. Zhang , C. J. Wei , X. M. Qi , Y. H. Li , X. L. Gao , Y. Wu , J. Neuroinflammat. 2023, 20, 293. 10.1186/s12974-023-02976-7 PMC10704691 38062440

[23] H. Y. Zhang , Y. Wang , Y. He , T. Wang , X. H. Huang , C. M. Zhao , L. Zhang , S. W. Li , C. Wang , Y. N. Qu , X. X. Jiang , J. Neuroinflammat. 2020, 17, 200.

[24] M. S. Finnin , J. R. Donigian , N. P. Pavletich , Nat. Struct. Biol. 2001, 8, 621. 11427894 10.1038/89668

[25] T. F. Pais , E. M. Szego , O. Marques , L. Miller‐Fleming , P. Antas , P. Guerreiro , R. M. de Oliveira , B. Kasapoglu , T. F. Outeiro , EMBO J. 2013, 32, 2603. 24013120 10.1038/emboj.2013.200 PMC3791374

[26] F. Yuan , Z. M. Xu , L. Y. Lu , H. Nie , J. Ding , W. H. Ying , H. L. Tian , J. Neurochem. 2016, 136, 581. 26546505 10.1111/jnc.13423

[27] M. I. Rosenbaum , L. S. Clemmensen , D. S. Bredt , B. Bettler , K. Stromgaard , Nat. Rev. Drug Discovery 2020, 19, 884. 33177699 10.1038/s41573-020-0086-4

[28] Z. Blumenfeld , K. Bera , E. Castren , H. A. Lester , Neuropsychopharmacology 2024, 49, 246. 37783840 10.1038/s41386-023-01725-x PMC10700606

[29] T. Lan , Y. Li , X. Chen , W. Wang , C. Wang , H. Lou , S. Chen , S. Yu , Adv. Sci. (Weinh) 2024, 12, 2408618. 39574315 10.1002/advs.202408618 PMC11744721

[30] J. W. Mo , P. L. Kong , L. Ding , J. Fan , J. Ren , C. L. Lu , F. Guo , L. Y. Chen , R. Mo , Q. L. Zhong , Y. L. Wen , T. T. Gu , Q. W. Wang , S. J. Li , T. Guo , T. M. Gao , X. Cao , Adv. Sci. (Weinh) 2024, 11, 2403389. 39264289 10.1002/advs.202403389 PMC11538709

[31] Y. Wu , J. Deng , J. Ma , Y. Chen , N. Hu , S. Hao , B. Wang , Adv. Sci. (Weinh) 2024, 11, 2402152. 38946585 10.1002/advs.202402152 PMC11434213

[32] V. Lewis , G. Rurak , N. Salmaso , A. Aguilar‐Valles , Trends Neurosci. 2024, 47, 195. 38220554 10.1016/j.tins.2023.12.004

[33] E. M. Hess , L. M. Riggs , M. Michaelides , T. D. Gould , Biochem. Pharmacol. 2022, 197, 114892. 34968492 10.1016/j.bcp.2021.114892 PMC8883502

[34] T. Rumpf , S. Gerhardt , O. Einsle , M. Jung , Acta Crystallogr. F‐Struct Biol. Commun. 2015, 71, 1498. 26625292 10.1107/S2053230X15019986 PMC4666478

[35] Schrödinger Release 2024‐4: Maestro, Schrödinger , LLC, New York, NY, 2024.

[36] O. Trott , A. J. Olson , J. Comput. Chem. 2010, 31, 455. 19499576 10.1002/jcc.21334 PMC3041641

[37] S. Jo , T. Kim , V. G. Iyer , W. Im , J. Comput. Chem. 2008, 29, 1859. 18351591 10.1002/jcc.20945

[38] D. Van Der Spoel , E. Lindahl , B. Hess , G. Groenhof , A. E. Mark , H. J. Berendsen , J. Comput. Chem. 2005, 26, 1701. 16211538 10.1002/jcc.20291

[39] J. Huang , S. Rauscher , G. Nawrocki , T. Ran , M. Feig , B. L. de Groot , H. Grubmüller , A. D. MacKerell Jr. , Nat. Methods 2017, 14, 71. 27819658 10.1038/nmeth.4067 PMC5199616

[40] D. A. Case , H. M. Aktulga , K. Belfon , D. S. Cerutti , G. A. Cisneros , V. W. D. Cruzeiro , N. Forouzesh , T. J. Giese , A. W. Götz , H. Gohlke , S. Izadi , K. Kasavajhala , M. C. Kaymak , E. King , T. Kurtzman , T. S. Lee , P. Li , J. Liu , T. Luchko , R. Luo , M. Manathunga , M. R. Machado , H. M. Nguyen , K. A. O'Hearn , A. V. Onufriev , F. Pan , S. Pantano , R. Qi , A. Rahnamoun , A. Risheh , et al., J. Chem. Inf. Model. 2023, 63, 6183. 37805934 10.1021/acs.jcim.3c01153 PMC10598796

[41] B. Hess , H. Bekker , H. J. C. Berendsen , J. G. E. M. Fraaije , J. Comput. Chem. 1997, 18, 1463.

[42] U. Essmann , L. Perera , M. L. Berkowitz , T. Darden , H. Lee , L. G. Pedersen , J. Chem. Phys. 1995, 103, 8577.

[43] M. Parrinello , A. Rahman , J. Appl. Phys. 1981, 52, 7182.

[44] S. Nosé , M. L. Klein , Mol. Phys. 1983, 50, 1055.

[45] S. Nosé , Mol. Phys. 1984, 52, 255.

[46] W. G. Hoover , Phys. Rev. A. 1985, 31, 1695. 10.1103/physreva.31.1695 9895674

[47] R. Gowers , M. Linke , J. Barnoud , T. Reddy , M. Melo , S. L. Seyler , D. Dotson , J. Domanski , S. Buchoux , I. Kenney , MDAnalysis: a Python Package for the Rapid Analysis of Molecular Dynamics Simulations, Los Alamos National Laboratory, Los Alamos, NM, USA 2016.

[48] C. Bouysset , S. Fiorucci , J. Cheminformat. 2021, 13, 72. 10.1186/s13321-021-00548-6 PMC8466659 34563256

[49] W. Humphrey , A. Dalke , K. Schulten , J. Mol. Graph. Model. 1996, 14, 33. 10.1016/0263-7855(96)00018-5 8744570

[50] W. L. DeLano , Proteins 2002, 30, 442.

