# Supplementary material for: S‐ketamine Alleviates Neuroinflammation and Attenuates Lipopolysaccharide‐Induced Depression Via Targeting SIRT2
Source: Adv Sci (Weinh). 2025 Apr 2;12(23):2416481. doi: 10.1002/advs.202416481 (PMC12199433; doi:10.1002/advs.202416481)
Supplement: Supplementary file 1 — Supporting Information [file ADVS-12-2416481-s002.docx]

Supporting Information for

**S-ketamine Alleviates Neuroinflammation and Attenuates Lipopolysaccharide-induced Depression via Targeting SIRT2**

*Cong Lin^#^, Xiaoxuan Zhou^#^, Mingqi Li, Cong Zhang, Haojiang Zhai, Haohong Li, Hongshuang Wang^*^, Xiaohui Wang^*^*

**This document includes:**

General chemistry methods and reagents………………….……….....…….Page S2

General procedure for the synthesis of 6-hydroxyketamine, KP1, and KP2…........

……....................………….......................…..……............................……...Page S3-S4

Chiral resolution of racemic ketamine……….………....................………...Page S5

Table S1..…..……......................…..…..…….....……....................................Page S6

Table S2..…..……......................…..……..…….....…....................................Page S7

Table S3..…..……......................…..……..…….....…....................................Page S8

Figure S1..…..……......................…..……......…..…….................................Page S9

Figure S2..…..……......................…..……..…….....…..................................Page S10

Figure S3..…..……......................…..……..…….....…..................................Page S11

^1^H, ^13^C NMR and MS Spectra………..................………........…............…...Page S12-S17

HPLC Spectra of racemic ketamine, S-ketamine, and R-ketamine..........…...Page S18-S19

**General chemistry methods and reagents.**

Chemicals and solvents were purchased from Energy Chemical (Shanghai, China) unless otherwise stated. All the commercial reagents and solvents were used as such without further purification. Analytical thin-layer chromatography (TLC) and silica gel were purchased from Qingdao Shuoyuan Silicone Technology Co., Ltd. Flash chromatography was purchased from Biotage. Nuclear magnetic resonance (NMR) spectra were recorded on a Bruker AV-300 spectrometer (300 MHz ^1^H, 75 MHz ^13^C) using CDCl_3_ solution. Chemical shifts (δ) are expressed in ppm recorded using the residual solvent as the internal reference (CDCl_3_: ^1^H 7.26 ppm, ^13^C 77.16 ppm) in all cases. Signal splitting patterns are described as chemical shift, multiplicity (s = singlet, d = doublet, t = triplet, m = multiplet, and br = broad), coupling constant in hertz (Hz), and integration. Electrospray (ES) mass spectra were recorded on a Waters QDa mass spectrometer run in the positive mode.

**General procedure for the synthesis of 6-hydroxyketamine, KP1, and KP2**

**Scheme S1**. Synthesis of the probes **KP1** and **KP2**.

**Synthesis of 6-hydroxyketamine**

Ketamine (237.7 mg, 1 mmol) was dissolved in anhydrous tetrahydrofuran (THF) (5 mL) under nitrogen and cooled to -78 °C. To above solution, KHMDS (2 M in THF, 1.5 mL, 3 mmol) was added dropwise and stirred for 30 min. The nitrogen atmosphere was replaced with oxygen and P(OMe)_3_ (355 μL, 3 mmol) was added gradually, maintaining the low temperature for 2 h. The reaction was monitored by thin-layer chromatography (TLC) until the reaction was completed. The mixture was quenched with saturated ammonium chloride aqueous (10 mL) and extracted with ethyl acetate (3 × 20 mL). The combined organic extracts were sequentially washed with saturated brine, dried over anhydrous Na_2_SO_4_ and filtered. The solvent was removed under a vacuum. The residue was purified by column chromatography on silica gel with EtOAc/petroleum ether to give 6-hydroxyketamine (201 mg, 79%). **^1^H NMR** (300 MHz, CDCl_3_) δ 7.49 (dd, *J* = 7.8, 1.4 Hz, 1H), 7.42-7.27 (m, 2H), 7.30 (dd, 1H, *J* = 7.5, 1.7 Hz,), 4.19 (dd, *J* = 11.4, 6.7 Hz, 1H), 3.08-3.00 (m, 1H), 2.39-2.30 (m, 1H), 2.08 (s, 3H), 1.79-1.41 (m, 4H). **^13^C NMR** (75 MHz, CDCl_3_) δ 212.46, 135.08, 134.38, 131.44, 129.68, 129.39, 126.78, 73.55, 70.62, 39.84, 38.67, 28.64, 19.15. MS (ESI) m/z calcd for C_13_H_16_ClNNaO_2_^+^ [M + Na]^+^ 276.08, found 275.99.

**Synthesis of KP1**

To a solution of 6-hydroxyketamine (50 mg, 0.2 mmol) in THF (5 mL) under nitrogen, 3-bromopropynyl (20 μL, 0.24 mmol), potassium tert-butoxide (44 mg, 0.4 mmol), and sodium iodide (60 mg, 0.4 mmol) were added at 0 °C, and then heated to 30 °C and stirred for 3 h until the reaction completed. The mixture was quenched with saturated ammonium chloride solution (5 mL) and extracted with ethyl acetate (3 × 20 mL). The combined organic extracts were sequentially washed with saturated brine, dried over anhydrous Na_2_SO_4_ and filtered. The solvent was removed under a vacuum. The residue was purified by column chromatography on silica gel to give **KP1** (26 mg, 45%). **^1^H NMR** (300 MHz, CDCl_3_) δ 7.55 (dd, *J* = 7.7, 1.8 Hz, 1H), 7.35 (dd, *J* = 7.6, 1.6 Hz, 1H), 7.30-7.16 (m, 2H), 6.03 (dd, *J* = 5.5, 2.8Hz, 1H), 4.59 (t, *J* = 2.5 Hz, 1H), 2.93-2.81 (m, 1H), 2.68-2.54 (m, 1H), 2.50 (t, *J* = 2.4 Hz, 1H), 2.43-2.38 (m, 1H), 2.37-2.31 (m, 1H), 2.27 (s, 3H), 2.11 (br s, 1H), 1.94-1.86 (m, 1H). **^13^C NMR** (75 MHz, CDCl_3_) δ 189.30, 147.89, 138.45, 132.80, 131.20, 129.12, 128.60, 126.64, 118.12, 78.17, 75.92, 67.20, 56.12, 33.80, 30.25, 20.63. MS (ESI) m/z calcd for C_16_H_17_ClNO_2_^+^ [M + H]^+^ 290.09, found 290.01.

**Synthesis of KP2**

To a solution of 6-hydroxyketamine (44 mg, 0.17 mmol) in DMF (5 mL) under nitrogen, 3-bromopropynyl (17.5 μL, 0.21 mmol), cesium carbonate (113 mg, 0.334 mmol), and sodium iodide (52 mg, 0.34 mmol) were added at 0 °C. Then the mixture was moved to room temperature and stirred for 6 h. After the reaction was complete (as confirmed by TLC), the mixture was quenched with saturated ammonium chloride solution (5 mL) and extracted with ethyl acetate (3 × 20 mL). The combined organic extracts were sequentially washed with saturated brine (30 mL), dried over anhydrous Na_2_SO_4_ and filtered. The solvent was removed under a vacuum. The residue was purified by column chromatography on silica gel to **KP2** (35 mg, 70%). **^1^H NMR** (300 MHz, CDCl_3_) δ7.48 (dd, *J* = 7.8, 1.4 Hz, 1H), 7.43-7.34 (m, 2H), 7.32 (dd, *J* = 7.4, 1.7 Hz, 1H), 4.31-4.22 (m, 1H), 3.79 (dd, *J* = 16.9, 2.3 Hz, 1H), 3.72 (br s, 1H), 3.15-3.03 (m, 2H), 2.42 (s, 3H), 2.32-2.25 (m, 1H), 2.02 (t, *J* = 2.4 Hz, 1H), 1.90-1.78 (m, 1H), 1.74-1.67 (m, 1H), 1.48-1.39 (m, 2H). **^13^C NMR** (75 MHz, CDCl_3_) δ 211.44, 135.26, 132.52, 131.71, 130.18, 129.82, 127.02, 80.82, 74.65, 74.33, 71.87, 41.28, 39.51, 37.99, 35.03, 19.00. MS (ESI) m/z calcd for C_16_H_18_ClNNaO_2_^+^ [M + Na]^+^ 314.09, found 314.03.

**Chiral resolution of racemic ketamine**

**Scheme S2.** Resolution of R-ketamine from racemic ketamine.

To a solution of (*rac*)-ketamine (200 mg, 0.84 mmol) in acetone (1226 μL), D-tartaric acid (126 mg, 0.84 mmol) was added and heated to reflux. Water (160 μL) was then added and refluxed for 10 min, after which the solution became clear. The mixture was allowed to cool naturally to room temperature and was left to stand overnight. The white solid was then filtered and dried to yield the crude product (197 mg). The crude product was subsequently recrystallized in a mixture of acetone (1226 μL) and water (90 μL) to yield (R)-ketamine-D-tartaric acid salt (102.5 mg, 63% yield).

The (R)-ketamine-D-tartaric acid salt was dissolved in saturated sodium carbonate aqueous (10 mL) and extracted with ethyl acetate (3 × 10 mL). The combined organic extracts were sequentially washed with saturated brine, dried over anhydrous Na_2_SO_4_ and filtered. The solvent was removed under a vacuum to give (R)-ketamine (62 mg). To a solution of (R)-ketamine in Et_2_O (5 mL), HCl-dioxane (4M, 200 μL) was added and stirred for 20 min. The white solid produced was filtered and dried to give (R)-ketamine-HCl (69 mg, [α]_D_^25^ = -93.5 (C = 1, H_2_O)).

**Table S1.** Summarized details of materials and reagents

| Reagents | Manufacturers | CAS |
| --- | --- | --- |
| Sodium carbonate | Xilong Scientific | 497-19-8 |
| D-(−)-Tartaric acid | Energy Chemical | 147-71-7 |
| Tetrahydrofuran | Energy Chemical | 109-99-9 |
| Ethyl acetate | Adamas | 141-78-6 |
| Sodium sulfate | Xilong Scientific | 7757-82-6 |
| 3-Bromopropyne | Energy Chemical | 106-96-7 |
| Potassium tert-butoxide | Energy Chemical | 865-47-4 |
| Sodium iodide | Energy Chemical | 7681-82-5 |
| N, N-Dimethylformamide | Energy Chemical | 68-12-2 |
| Cesium carbonate | Energy Chemical | 534-17-8 |
| Potassium bis(trimethylsilyl)amide | Energy Chemical | 40949-94-8 |
| Ammonium chloride | Adamas | 12125-02-9 |

Table S2. Primer sequences of IL-1β, TNF-α, SIRT2 and Rpl27

| Gene |  | Sequence (5’-3’) |
| --- | --- | --- |
| IL-1β | Forward | CCACCTTTTGACAGTGATGA |
|  | Reverse | GAGATTTGAAGCTGGATGCT |
| TNF-α | Forward | CCCTCCAGAAAAGACACCATG |
|  | Reverse | GCCACAAGCAGGAATGAGAAG |
| Rpl27 | Forward | AAGCCGTCATCGTGAAGAACA |
|  | Reverse | CTTGATCTTGGATCGCTTGGC |
| SIRT2 | Forward | CCTGGCTGGGTGACTGTGAT |
|  | Reverse | GTGACCCTGACTGGGCATCTAT |

Table S3. Primer sequences of mock and SIRT2 short hairpin RNAs

| Gene |  | Sequence (5’-3’) |
| --- | --- | --- |
| Mock shRNA | Forward | CCGGAATGCCTACGTTAAGCTATACCTCGAGGTATAGCTTAACGTAGGCATTTTTTTG |
|  | Reverse | AATTCAAAAAAATGCCTACGTTAAGCTATACCTCGAGGTATAGCTTAACGTAGGCATT |
| shSIRT2#1 | Forward | CCGGCTTACCCAGAGGCCATCTTTGCTCGAGCAAAGATGGCCTCTGGGTAAGTTTTTG |
|  | Reverse | AATTCAAAAACTTACCCAGAGGCCATCTTTGCTCGAGCAAAGATGGCCTCTGGGTAAG |
| shSIRT2#2 | Forward | CCGGCCTCTATGCAAACCTGGAGAACTCGAGTTCTCCAGGTTTGCATAGAGGTTTTTG |
|  | Reverse | AATTCAAAAACCTCTATGCAAACCTGGAGAACTCGAGTTCTCCAGGTTTGCATAGAGG |


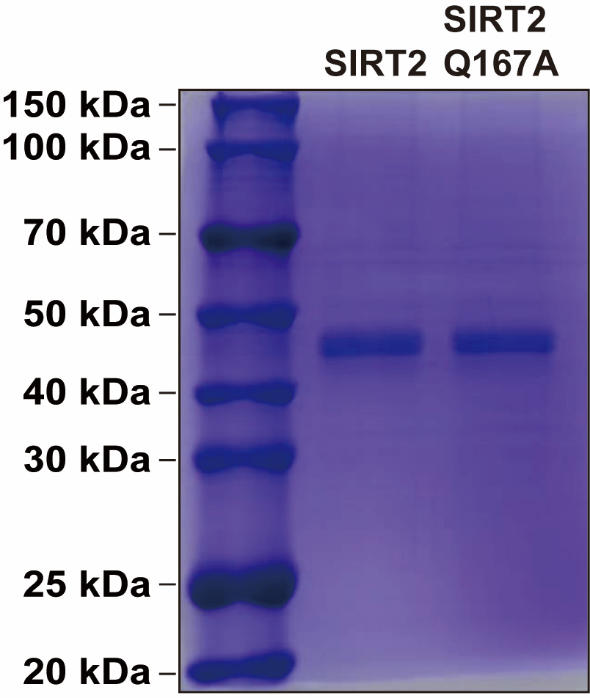


**Figure S1.** Coomassie blue staining images of the recombinant His-SIRT2 and His-SIRT2 Q167A proteins.


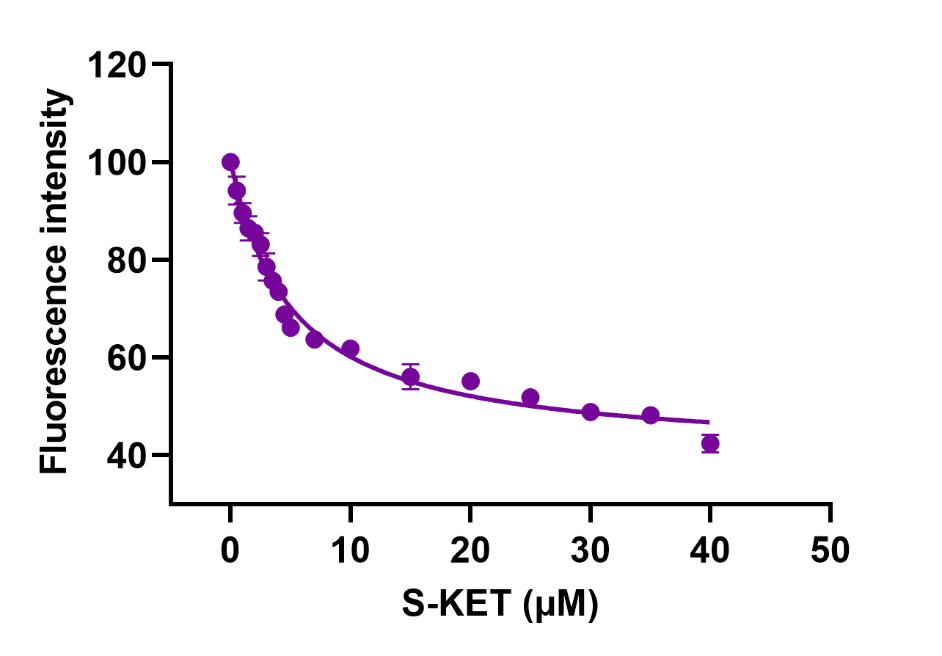


**Figure S2.** The titration curve of SIRT2 intrinsic fluorescence intensity with increasing S-KET yielded a dissociation constant of 4.7 ± 0.5 μM. The experiments were performed in triplicate, with data presented as mean ± SEM.


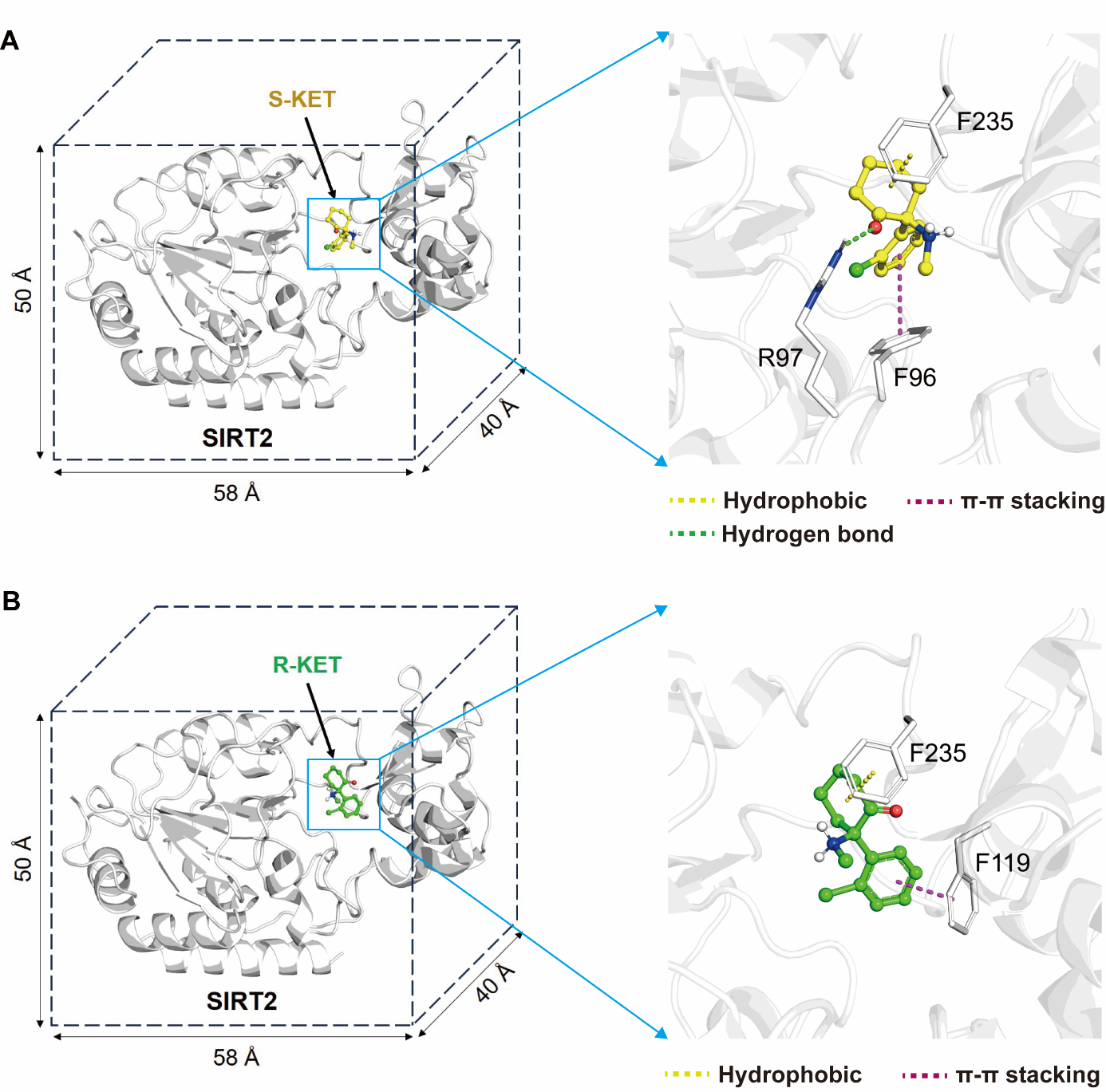


**Figure S3.** Diagrams illustrating the optimal binding modes of S-ketamine (A) (yellow, ball-and-stick) and R-ketamine (B) (green, ball-and-stick) to SIRT2 (silver, cartoon), as determined by molecular docking. The black dashed line represents the position and dimensions of the docking box.

**^1^H, ^13^C NMR and MS Spectra**


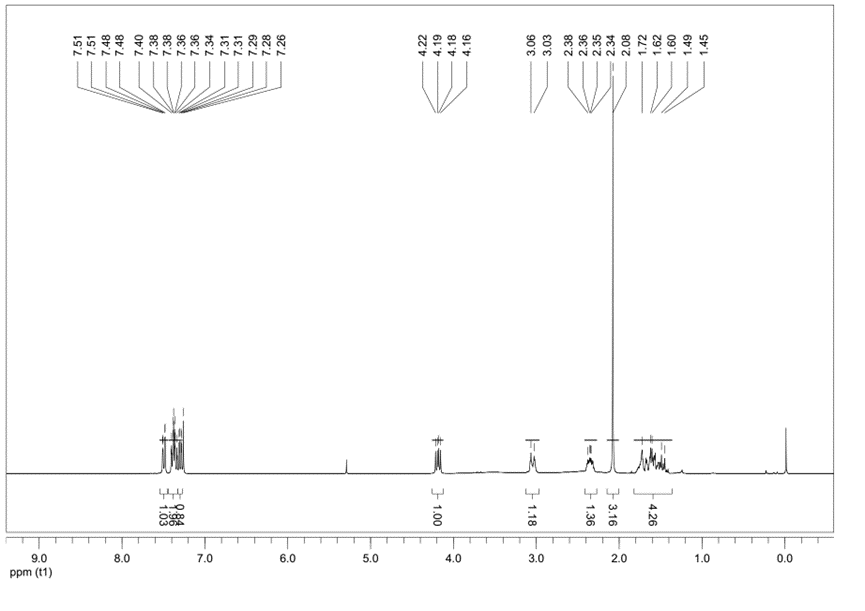


^1^H NMR spectrum of 6-hydroxyketamine


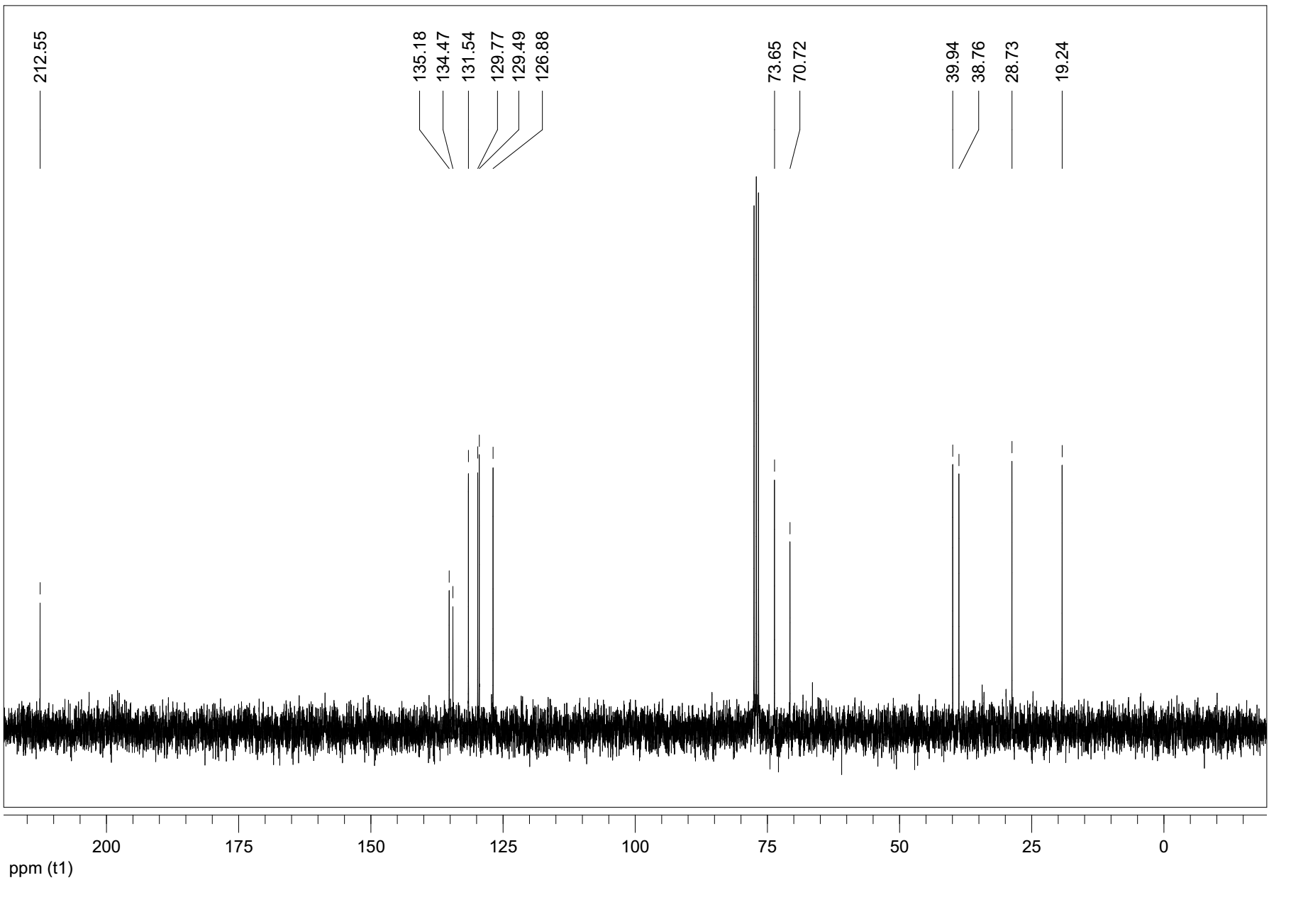


^13^C NMR spectrum of 6-hydroxyketamine


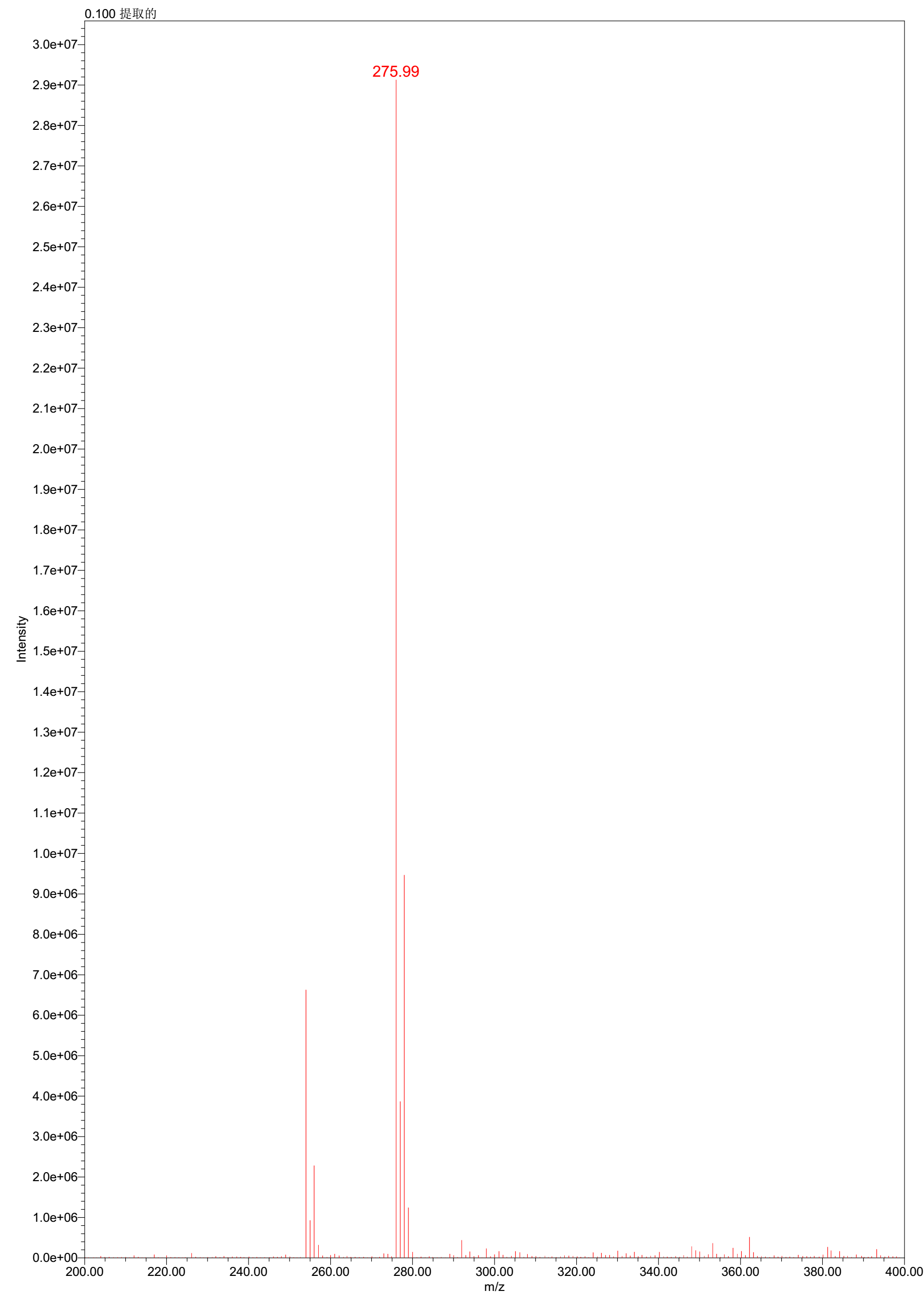


MS spectrum of 6-hydroxyketamine


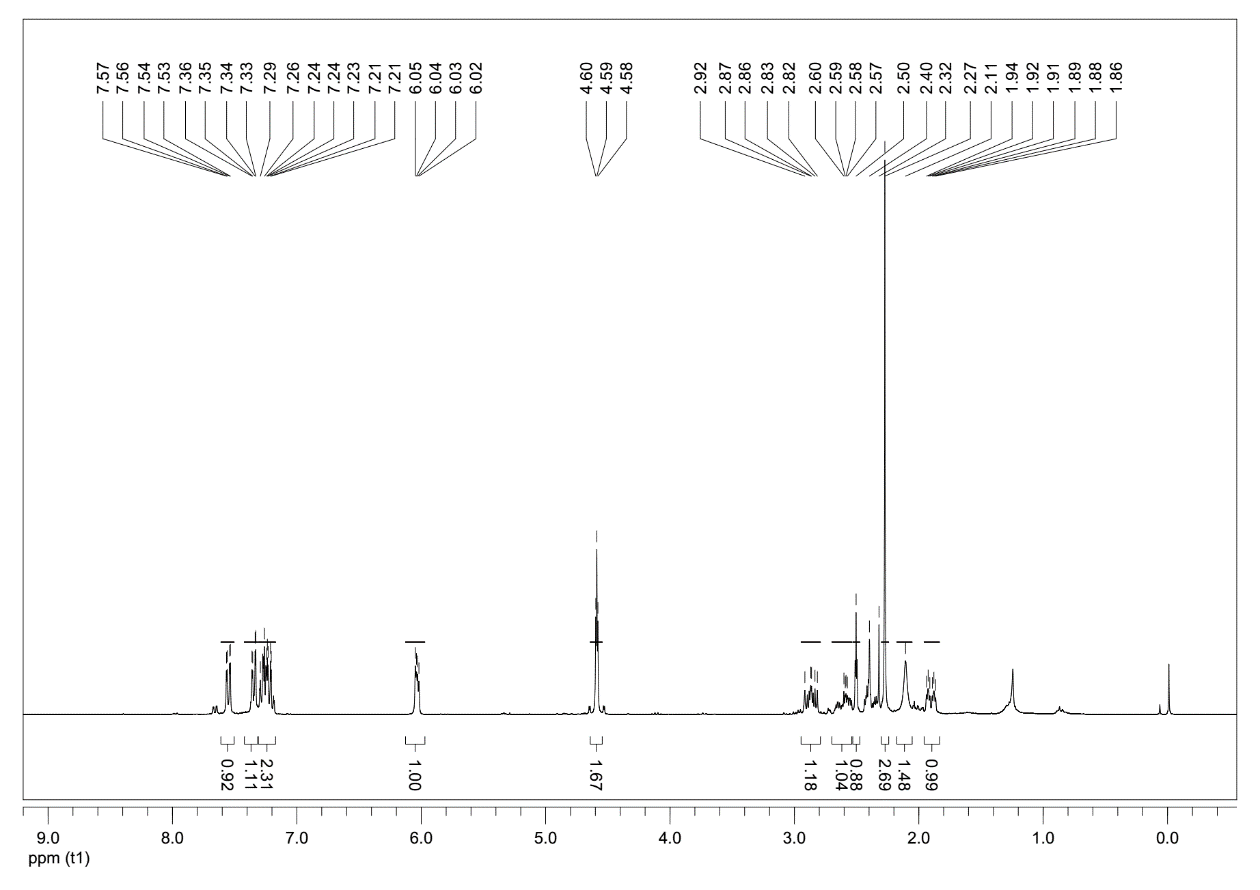


^1^H NMR spectrum of **KP1**


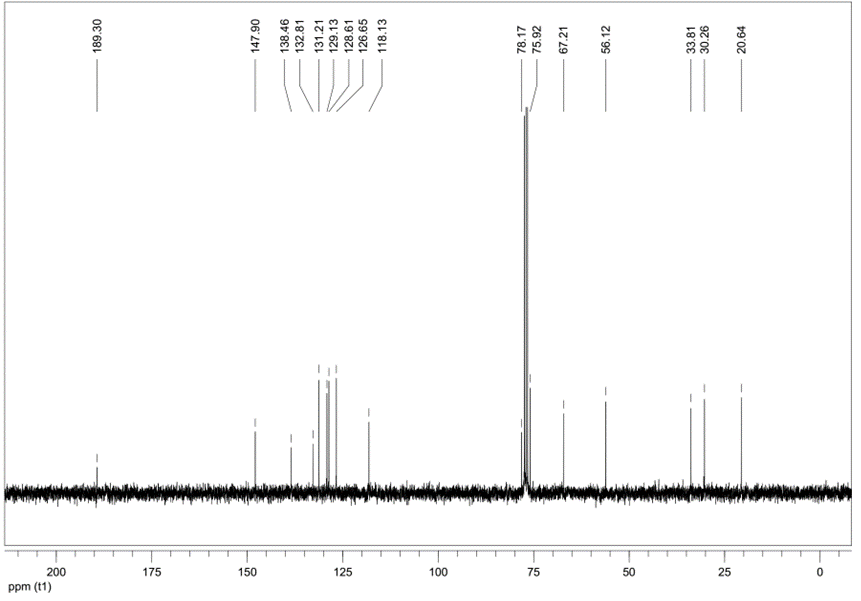


^13^C NMR spectrum of **KP1**


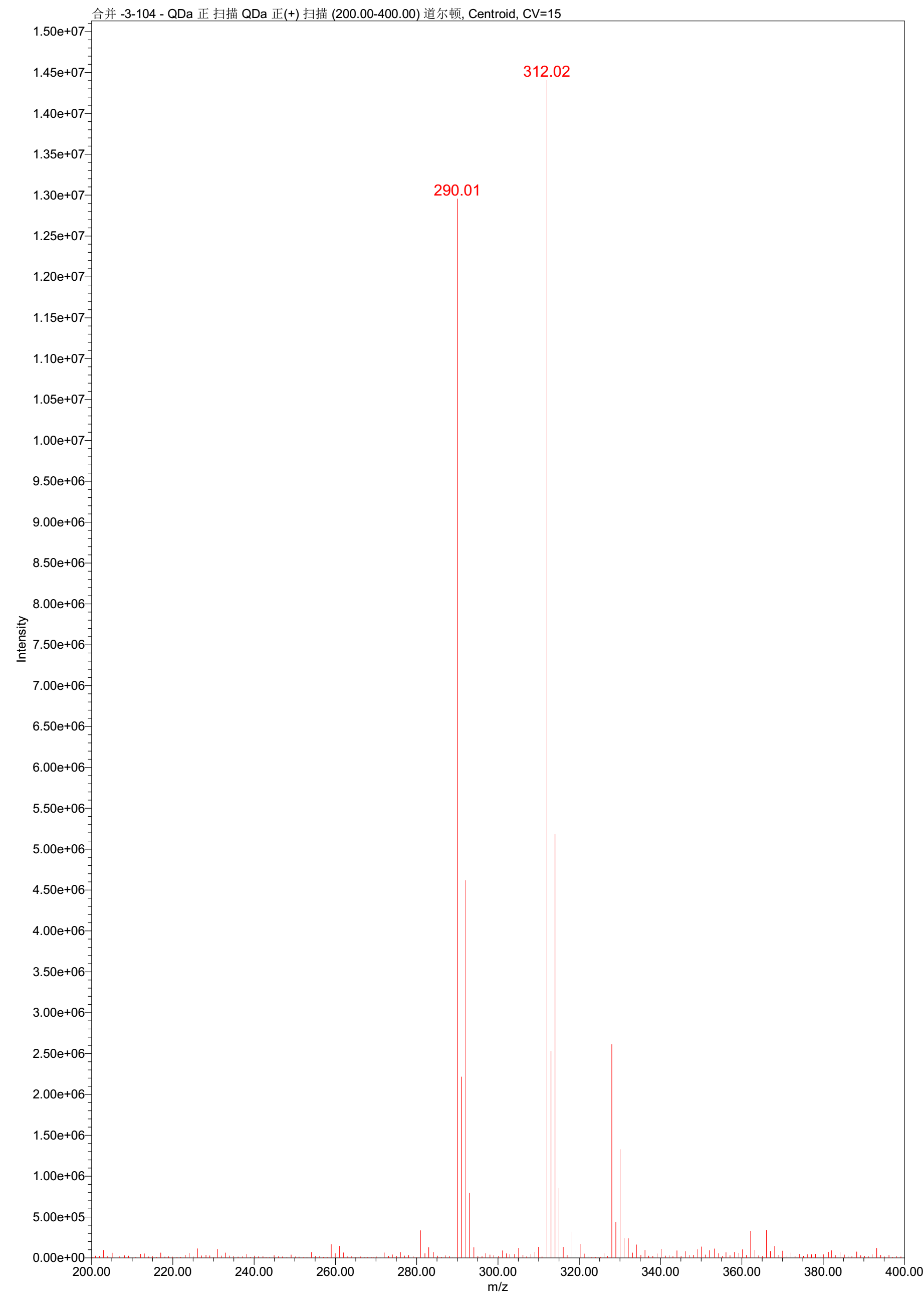


MS spectrum of **KP1**


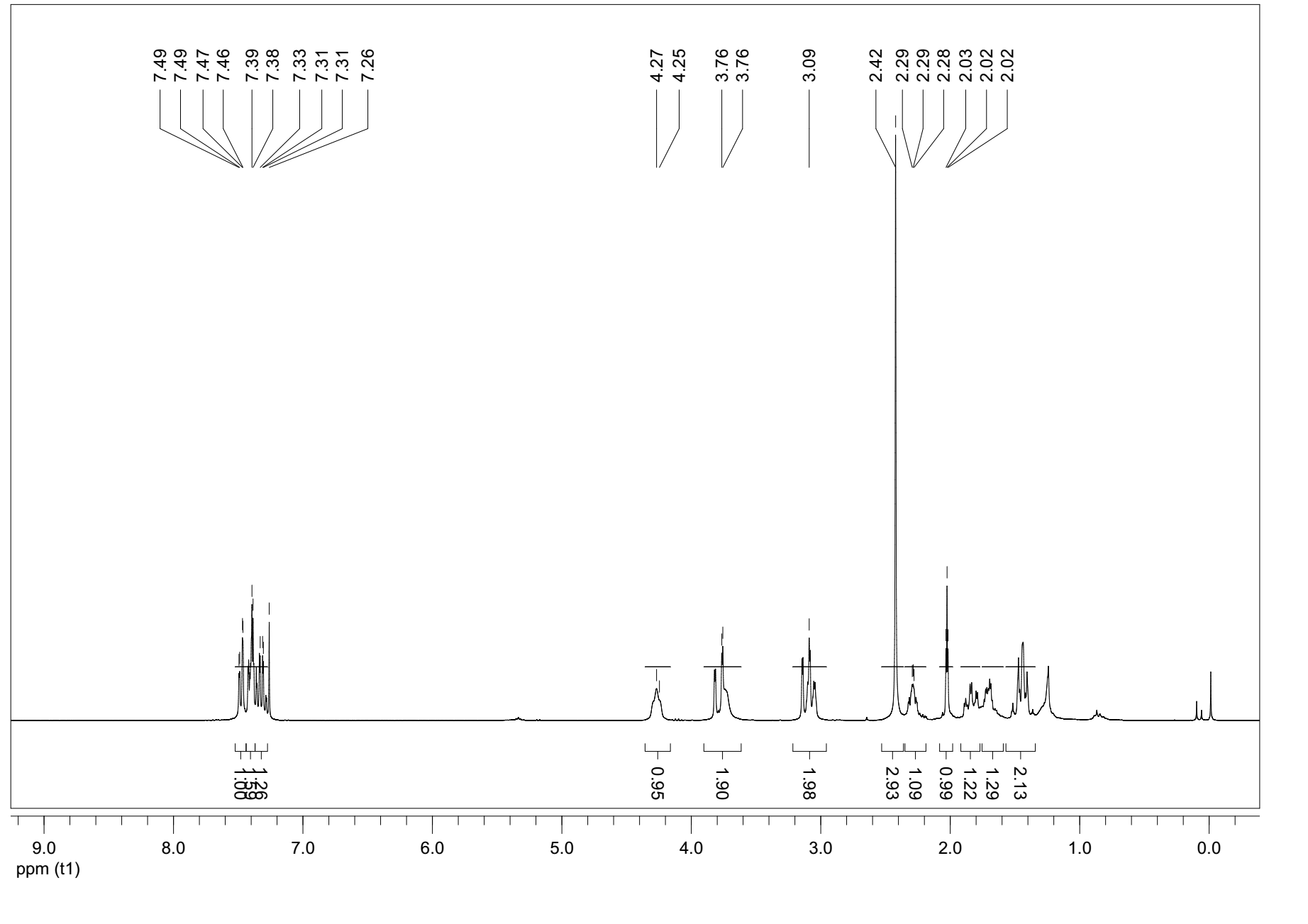


^1^H NMR spectrum of **KP2**


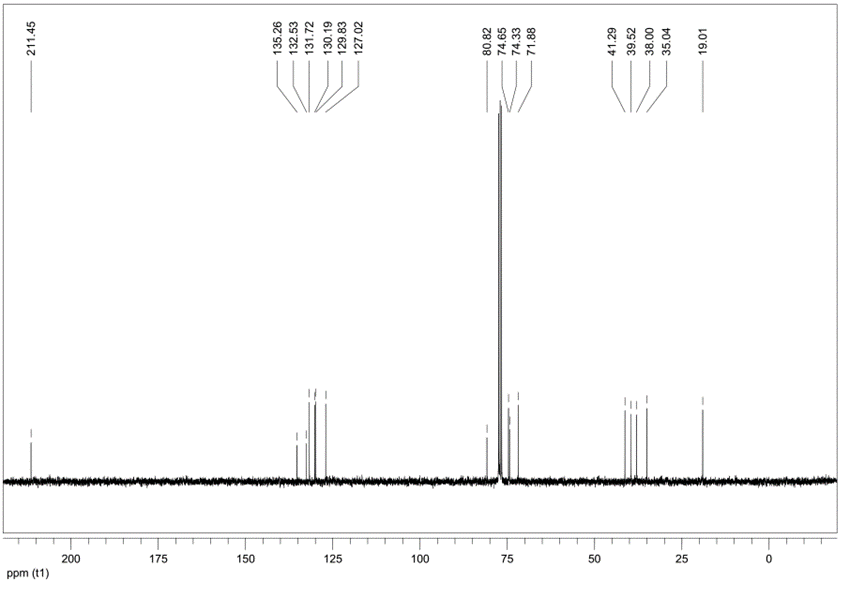


^13^C NMR spectrum of **KP2**


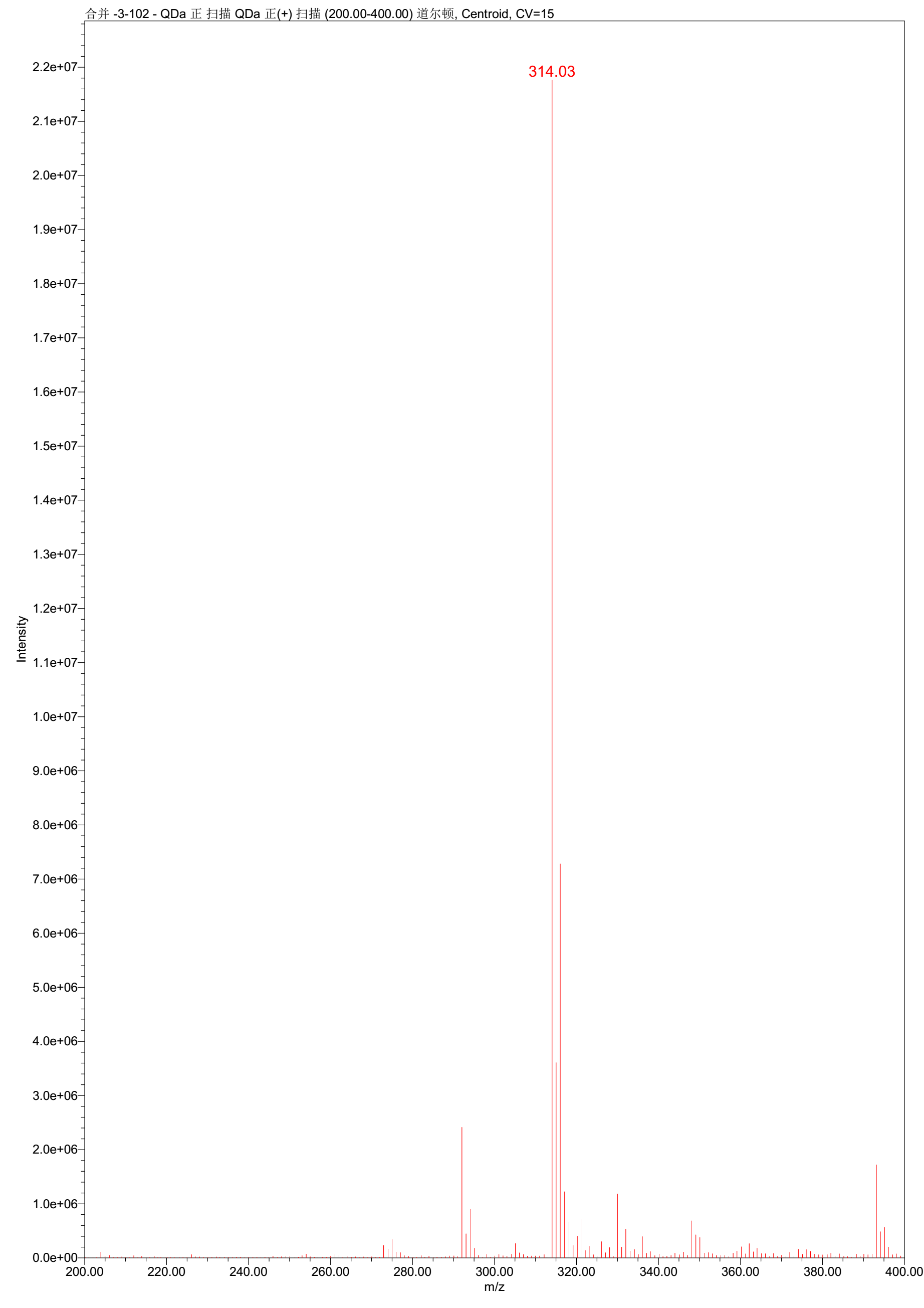


MS spectrum of **KP2**

**racemic-ketamine**

| **Detector A (210 nm)** | | |  |  |  |  |  |
| --- | --- | --- | --- | --- | --- | --- | --- |
| **Pk #** | **Retention Time** | **Area** | | | **Area %** | **Height** | **Height %** |
| 1 | 9.398 | 50340624 | | | 45.052 | 1879039 | 51.653 |
| 2 | 12.757 | 61397257 | | | 54.948 | 1758795 | 48.347 |
| Totals |  | 111737882 | | | 100.000 | 3637833 | 100.000 |

**HPLC**: chiralcel AS-H column (hexane/isopropanol = 95/5, flow rate = 1.0 mL/min, UV = 210 nm), t_S_:9.398 min, t_R_:12.757 min.

**S-ketamine**

| **Detector A (210 nm)** | | |  |  |  |  |  |
| --- | --- | --- | --- | --- | --- | --- | --- |
| **Pk #** | **Retention Time** | **Area** | | | **Area %** | **Height** | **Height %** |
| 1 | 9.511 | 47002502 | | | 100.000 | 1857972 | 100.000 |
| Totals |  | 47002502 | | | 100.000 | 1857972 | 100.000 |

**HPLC**: chiralcel AS-H column (hexane/isopropanol = 95/5, flow rate = 1.0 mL/min, UV = 210 nm), t_S_:9.511 min, 100% *ee*.

**R-ketamine**

| **Detector A (210 nm)** | | |  |  |  |  |  |
| --- | --- | --- | --- | --- | --- | --- | --- |
| **Pk #** | **Retention Time** | **Area** | | | **Area %** | **Height** | **Height %** |
| 1 | 12.896 | 39736020 | | | 100.000 | 1400539 | 100.000 |
| Totals |  | 39736020 | | | 100.000 | 1400539 | 100.000 |

**HPLC**: chiralcel AS-H column (hexane/isopropanol = 95/5, flow rate = 1.0 mL/min, UV = 210 nm), t_R_:12.896 min, 100% *ee*.
